# Molecular insight into thiopurine resistance: transcriptomic signature in lymphoblastoid cell lines

**DOI:** 10.1186/s13073-015-0150-6

**Published:** 2015-04-18

**Authors:** Laurent Chouchana, Ana Aurora Fernández-Ramos, Florent Dumont, Catherine Marchetti, Irène Ceballos-Picot, Philippe Beaune, David Gurwitz, Marie-Anne Loriot

**Affiliations:** INSERM UMR-S 1147, 45 rue des Saints-Pères, Paris, 75006 France; Université Paris Descartes, Sorbonne Paris Cité, 45 rue des Saints-Pères, Paris, 75006 France; INSERM U1016, Institut Cochin, 22 Rue Mechain, Paris, 75014 France; Assistance Publique-Hôpitaux de Paris, Hôpital Européen Georges Pompidou, Biochimie Pharmacogénétique et Oncologie Moléculaire, 20 rue Leblanc, Paris, 75015 France; Assistance Publique-Hôpitaux de Paris, Hôpital Necker-Enfants Malades, Biochimie Métabolique, 149 Rue de Sèvres, Paris, 75015 France; Department of Human Molecular Genetics and Biochemistry, Sackler School of Medicine, Tel-Aviv University, Tel-Aviv, Israel

## Abstract

**Background:**

There has been considerable progress in the management of acute lymphoblastic leukemia (ALL) but further improvement is needed to increase long-term survival. The thiopurine agent 6-mercaptopurine (6-MP) used for ALL maintenance therapy has a key influence on clinical outcomes and relapse prevention. Genetic inheritance in thiopurine metabolism plays a major role in interindividual clinical response variability to thiopurines; however, most cases of thiopurine resistance remain unexplained.

**Methods:**

We used lymphoblastoid cell lines (LCLs) from healthy donors, selected for their extreme thiopurine susceptibility. Thiopurine metabolism was characterized by the determination of TPMT and HPRT activity. We performed genome-wide expression profiling in resistant and sensitive cell lines with the goal of elucidating the mechanisms of thiopurine resistance.

**Results:**

We determined a higher TPMT activity (+44%; *P* = 0.024) in resistant compared to sensitive cell lines, although there was no difference in HPRT activity. We identified a 32-gene transcriptomic signature that predicts thiopurine resistance. This signature includes the *GTPBP4* gene coding for a GTP-binding protein that interacts with p53. A comprehensive pathway analysis of the genes differentially expressed between resistant and sensitive cell lines indicated a role for cell cycle and DNA mismatch repair system in thiopurine resistance. It also revealed overexpression of the ATM/p53/p21 pathway, which is activated in response to DNA damage and induces cell cycle arrest in thiopurine resistant LCLs. Furthermore, overexpression of the p53 target gene *TNFRSF10D* or the negative cell cycle regulator *CCNG2* induces cell cycle arrest and may also contribute to thiopurine resistance. *ARHGDIA* under-expression in resistant cell lines may constitute a novel molecular mechanism contributing to thiopurine resistance based on Rac1 inhibition induced apoptosis and in relation with thiopurine pharmacodynamics.

**Conclusion:**

Our study provides new insights into the molecular mechanisms underlying thiopurine resistance and suggests a potential research focus for developing tailored medicine.

**Electronic supplementary material:**

The online version of this article (doi:10.1186/s13073-015-0150-6) contains supplementary material, which is available to authorized users.

## Background

Approximately 6,000 patients are diagnosed with acute lymphoblastic leukemia (ALL) each year in the USA, including about two-thirds who are younger than 20 years, making ALL the most common malignancy in children and adolescents [1]. Clinical outcomes of childhood ALL have improved considerably over time, and the overall 5-year event-free survival rate now reaches 80%, and even 90% when treatment strategies are selected based on the biological features of the leukemic cells and the pharmacodynamic and pharmacogenomic characteristics of the patient [1-3]. Nevertheless, this leaves about 20% of patients who experience relapses with far lower survival rates that decrease with each relapse [4]. Furthermore, the outcomes are less favorable in adults, whose complete recovery rates rarely exceed 40% and who exhibit greater resistance to, and poorer tolerance of, therapeutic agents compared to children [2].

The treatment of ALL relies on combination chemotherapy. One of the cornerstone drugs for both intensification and maintenance therapy is the thiopurine agent 6-mercaptopurine (6-MP) [2]. Effective maintenance therapy is essential for stabilizing the remission by suppressing the re-emergence of drug-resistant clones via a continuous reduction in the burden of residual leukemic cells. Recent studies have shown that, even when multiple drugs are used, the response to single drugs exerts a major influence on the relapse risk and that 6-MP is among the drugs with the greatest influence on remission maintenance [5]. Therefore, elucidating the molecular basis of 6-MP resistance is crucial to relapse prediction, which allows optimization of the treatment strategy [6].

Genetic inheritance in thiopurine metabolism plays a major role in the interindividual variability that characterizes the clinical response to thiopurine agents [7,8]. Extensive pharmacogenetic studies have focused on the enzymes involved in thiopurine metabolism, such as thiopurine *S*-methyltransferase (TPMT), hypoxanthine phosphoribosyltransferase (HPRT), and inosine triphosphate pyrophosphatase (ITPA). These studies have shed light on the toxicity of thiopurine agents but have provided little information on thiopurine resistance [7-10]. To date, except rare cases of HPRT-deficiency or ultra-high TPMT activity, which impair the production of active thiopurine metabolites, most cases of thiopurine resistance remain unexplained [11-16].

Transcriptomic analysis is a powerful tool for characterizing susceptibility and resistance to drugs [17,18]. This approach can uncover previously unrecognized mechanisms of drug response and provides information on the associated biological pathways. Lymphoblastoid cell lines (LCLs) constitute a well-established pharmacogenomic model for genome-wide expression profiling [18,19]. Although there has been some debate about biological noise related to confounding factors, LCLs have been used to assess gene sets involved in responses to anticancer drugs such as bleomycin, gemcitabine, cytosine arabinoside, and 5-fluorouracil [20-24]. A gene set analysis of a vast panel of LCLs from different ethnic groups was conducted to assess associations between basal gene expression and thiopurine susceptibility [22]. The 3′,5′-cyclic-AMP phosphodiesterase activity and the γ-aminobutyric acid catabolic process were found to be involved in the thiopurine response [22]. However, this study was designed to evaluate gene expression profiles associated with a broad range of thiopurine susceptibility levels rather than with thiopurine resistance.

Here, we investigated the molecular basis of thiopurine resistance by exploring the whole-genome basal transcriptomic profiles involved in 6-MP resistant and sensitive phenotypes of LCLs originating from unrelated healthy individuals and selected by *in vitro* growth inhibition assays. We then used these transcriptomic profiles to identify genes predicting thiopurine resistance and relevant 6-MP metabolic pathways.

## Methods

### Cell lines

We screened 53 LCLs originating from consenting unrelated healthy adults and obtained via a collaboration program with the National Laboratory for the Genetics of Israeli Populations (NLGIP), Tel-Aviv University, Israel [25,26]. Six additional LCLs from male patients with the recessive genetic disease Lesch-Nyhan syndrome were obtained from the biobank of the Biochemistry and Molecular Biology Department of the Cochin University Hospital, Paris, France. They served as positive phenotypic controls for thiopurine resistance, as Lesch-Nyhan syndrome is characterized by HPRT deficiency. Cells were cultured as described elsewhere [25].

### Ethical conduct of research

The authors state that they have obtained appropriate institutional review board approval for the collection of these samples in accordance with local legislation, or have followed the ethical principles outlined in the Declaration of Helsinki for experimental investigations. In addition, informed written consent has been obtained from the healthy donors and patients, or their guardians, involved in this study.

## Material

Roswell Park Memorial Institute medium, L-glutamine, and antibiotics for cell culture were purchased from Life Technologies (Carlsbad, CA, USA), fetal bovine serum from GE Healthcare (Little Chalfont, UK), and Falcon cell culture materials from Fisher Scientific (Waltham, MA, USA). Drugs (6-MP, azathioprine and 6-thioguanine [6-TG]) were purchased from Sigma-Aldrich (St. Louis, MO, USA). Stock solutions (concentration, 5 mM) were prepared in 0.1 N sodium hydroxide and diluted in phosphate-buffered saline (PBS) to working solutions containing at least 25-fold the final tested concentrations.

### Cell proliferation assay

Drug concentrations for assessing growth inhibition were 2 μM for 6-MP, 5 μM for azathioprine, and 0.5 μM for 6-TG. These concentrations were close to the mean half-maximal inhibitory concentration (IC_50_) and allowed optimal LCL classification [25]. The cells were diluted to 200,000 /mL then incubated in Falcon 96-well plates (Waltham, MA, USA) in a volume of 200 μL (40,000 cells/well) for 3 days, with drug working solutions added as needed in three replicates, and six replicates for the controls (cells treated with 20 μL PBS). After 72 h, the tetrazolium derivative MTS reagent (CellTiter 96® AQueous One Solution Cell Proliferation Assay, Promega, Madison, WI, USA) was added (volume, 40 μL) to each well, including blank wells containing only PBS solution. After further incubation for 4 h, absorption at 490 nm was measured using a microplate reader spectrophotometer (Safir™ Tecan, Männedorf, Switzerland), which is ascribable to the living cells present in the medium. Growth inhibition relative to control was assessed for each cell line at least twice on two different batches from different cell vials thawed from the liquid nitrogen stock. Reproducibility of drug susceptibility for repeated thawing cycles was high, as previously reported [25].

### Basal cell growth rate

Basal cell growth rates were estimated for each cell line during phenotyping experiments. After 72 h of the proliferation assay, cell density (*N*_*t*_) was estimated in PBS control wells using MTS reagent, as described above, and calibration curve. The basal cell growth rate (*r*) was calculated using the following formula appropriate for the usual exponential kinetics of cell growth after a defined time (*t*): *N*_*t*_ = *N*_0_.2^*tr*^.

### Nucleic acid extraction and quantification

Nucleic acids were extracted from cells incubated under optimal growth conditions with no added drugs. DNA and total RNA were extracted from cell pellets using QiAmp® DNA miniKit and miRNeasy® miniKit (Qiagen, Venlo, The Netherlands), respectively, according to the manufacturer’s instructions. DNA was quantified using an ND-1000 spectrophotometer (Nanodrop technologies, Wilmington, DE, USA). RNA quality and quantity were assessed using the 2100-Bioanalyzer (Agilent Technologies, Santa Clara, CA, USA).

### Microarray experiment

After validation of RNA quality (RIN score ≥8), 50 ng of total RNA was reverse-transcribed using the Ovation PicoSL WTA System V2 (NuGEN Technologies, West Cumbria, UK), according to the manufacturer’s instructions. Biotin-labelled cDNA was then hybridized to GeneChip® Human Gene 2.0ST microarrays (Affymetrix, Santa Clara, CA, USA) at 45°C for 17 h. The microarrays were washed on the fluidic station FS450 according to the specific manufacturer’s protocols and scanned using the GCS3000 7G (Affymetrix). The scans were then analyzed with Expression Console software (Affymetrix) to obtain raw data (.cel files) and metrics for quality controls. Examination of these quality-control metrics and of raw-data distribution showed no outlier samples.

Data were normalized using the Robust Multi-array Average (RMA) algorithm in R software with the custom chip description file (CDF) version 17.0.0 [27]. Data are available on the NCBI Gene Expression Omnibus (GEO) via the accession number GSE61905 [28].

Differentially expressed gene enrichment analysis was carried out using the DAVID bioinformatics resources (NIH), based on gene ontology (GO) biological processes, and Ingenuity Pathways Analysis (Ingenuity® Systems, USA) [29-31]. To identify a transcriptomic signature predicting thiopurine resistance, we applied the ‘nearest shrunken centroids’ method using the Prediction Analysis of Microarrays (PAM) R package, which identifies predictive classifier genes [32].

### Microfluidic-based RT-qPCR assay

Microfluidic-based quantitative PCR assay was performed to validate the differential microarray expression patterns of the molecular signature genes. Transcripts were quantified for 40 genes of interest and four reference genes (*GUSB*/*GAPDH*/*RPL13A*/*B2M*). The RT-qPCR assay is detailed in the Additional file 1: Data S1 and Table S2.

### Intracellular ATP assay

Intracellular ATP was assayed on the day the cell proliferation assay was performed. Cell samples were kept on ice until the assay, as ATP is unstable. The assay was performed using the ATP Bioluminescence Assay Kit HS II (Roche, Germany) according to the manufacturer’s instructions. Briefly, luminescence measured in the microplate wells was related to intracellular ATP after the addition of luciferase and D-luciferin. Intracellular ATP concentrations were calculated using a calibration curve.

### EBV and mtDNA copy number

Copy number variations (CNVs) of Epstein-Barr virus (EBV) and mitochondrial (mt) DNA were determined in the LCL DNA samples using TaqMan® CNV Assays (Life Technologies, Carlsbad, CA, USA). Custom TaqMan® assays were designed using Primer 3; primer and probe sequences are reported in Additional file 1: Table S3. The EBV CNV assay interrogated a 66-bp fragment at the DNA polymerase locus multiplexed with an 87-bp fragment assay from RNAse P (*RPPH1* gene) as an internal reference, using the VIC® dye-labeled TAMRA™ probe and sequence-specific forward and reverse primers (Applied assay ID 4403328). The mtDNA copy number assay examined a 72-bp fragment at the *ND2* locus multiplexed with a 90-bp fragment assay from *NRF1* gene as an internal reference. Final concentrations for EBV primers, EBV probe, mtDNA primers, mtDNA probe, *NRF1* primers and *NRF1* probe were 90 nM, 250 nM, 30 nM, 250 nM, 900 nM, and 250 nM, respectively. RT-qPCR TaqMan® reactions were performed in 384-well plates (reaction volume, 10 μL) using 10 ng of DNA template (concentration, 5 ng/μL) and 5 μL of TaqMan® Genotyping Master Mix, according to the manufacturer’s protocol. Equal efficiency of amplification was observed for each assay in the multiplex reaction.

Gene CNVs were determined using the previously described 2^-ΔΔCt^ method [33]. The absolute EBV copy number was related to a calibrator DNA template from the Burkitt lymphoma-derived Namalwa cell line (ECACC, UK), which was determined by fluorescence *in-situ* hybridization to have integrated EBV copies in the diploid genome [34]. The relative mtDNA copy number was related to the sample having the highest Ct (that is, the lowest copy number).

### Intracellular enzymatic activities

#### TPMT activity

TPMT activity was assessed using the previously described reverse-phase high-performance liquid chromatography (HPLC) method therefore adapted for lymphocyte pellets [35]. The pellets were lysed by two freezing/thaw cycles in 200 μL PBS. Assay results are reported in pmol of 6-methylmercaptopurine formed per hour and per mg of total protein.

#### HPRT activity

HPRT activity was measured as the rate of inosine monophosphate (IMP) production, using the Precice® kit (Novocib, Lyon, France). Briefly, as described elsewhere, the assay is based on the effect of IMP-dehydrogenase, which catalyzes the irreversible oxidation of IMP to xanthosine monophosphate while simultaneously reducing NAD to NADH_2_, whose production is monitored directly at 340 nm using a microplate reader spectrophotometer [36]. Assay results are reported in nmol of IMP formed per hour and per mg of total protein.

### Statistical analysis

Data are described as mean ± standard error (SEM). To compare 6-MP resistant and sensitive cell lines, we used the non-parametric Mann–Whitney *t* test. Correlations were assessed using the Spearman *r*_*s*_ test.

Gene expression values were analyzed using unsupervised hierarchical clustering and principal component analysis (PCA). Then, to identify differentially expressed genes, we performed the parametric Student *t* test and computed fold-changes for the expression of each gene in thiopurine resistant versus sensitive cell lines. Genes associated with *P* values <0.01 were selected for functional bioinformatics analyses using DAVID and Ingenuity®. Statistical analyses were carried out using Partek® Genomics Suite™ (Partek Inc., St. Louis, MO, USA) and Prism 5.0 (GraphPad, San Diego, CA, USA).

## Results

### LCL selection and characterization

**Figure 1:**
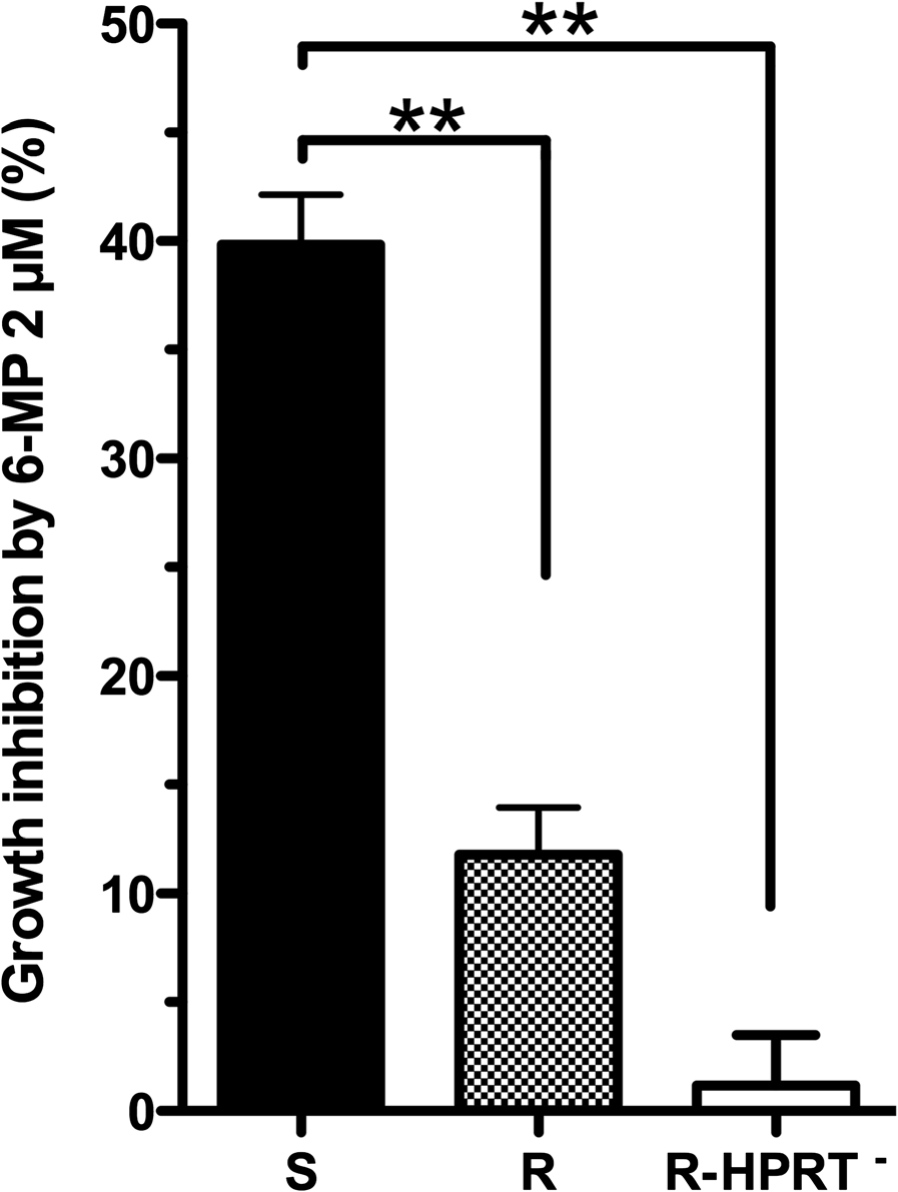
Growth inhibition by 6-MP (2 μM) in sensitive, resistant, and HPRT-deficient cell lines. **Mann Whitney test, *P* <0.01.

The relative susceptibility to growth inhibition by thiopurine drugs was determined for 53 LCLs from healthy adults. Growth inhibition by 6-MP was closely correlated to growth inhibition by azathioprine and by 6-TG (*r*_*s*_ = 0.95 and *r*_*s*_ = 0.81, respectively; *P* <0.0001) (Additional file 2: Figure S1). Growth inhibition by 6-MP was about 18 times greater for the most sensitive than for the most resistant cell lines (10th to 90th percentile growth inhibition, 12.4% to 42.6%). We performed a genome-wide expression analysis of 11 cell lines with an extreme phenotype in terms of 6-MP susceptibility, selected among the 53 LCLs: five resistant and six sensitive, with mean growth inhibition by 6-MP of 11.8 ± 2.1% and 39.8 ± 2.3%, respectively (*P* = 0.008) (Figure 1). The LCLs from the six patients with Lesch-Nyhan syndrome were almost completely resistant to thiopurines (mean growth inhibition by 6-MP, 1 ± 2%) (Figure 1). We verified that these cell lines carrying an inherited HPRT deficiency exhibited undetectable HPRT activity (mean: 0.2 ± 2 nmol/h/mg protein).

Resistant cell lines showed a trend toward a lower basal growth rate per day compared to sensitive cell lines (0.3 ± 0.06 *vs*. 0.5 ± 0.04, respectively; *P* = 0.052). Moreover, TPMT activity was about 44% higher in resistant compared to sensitive cell lines (425 ± 20 *vs*. 295 ± 24 pmol/h/mg protein; *P* = 0.024). None of the other study parameters (EBV copy number, mtDNA copy number, intracellular ATP level, and HPRT activity) differed significantly between 6-MP resistant and sensitive cell lines (Table 1).

**Table 1:** **Characteristics of lymphoblastoid cell lines**

	**Resistant (n = 5)**	**Sensitive (n = 6)**	***P***
Growth inhibition by 6-MP (%)	11.8 ± 2.1	39.8 ± 2.3	0.008
Basal growth rate per day	0.3 ± 0.06	0.5 ± 0.04	0.052
EBV copy number (absolute)	110 ± 35	47 ± 11	0.17
mtDNA copy number (relative)	1.5 ± 0.2	2.4 ± 0.3	0.08
Intracellular ATP level (μmol/10^6^ cells)	23.9 ± 2.6	19.0 ± 1.8	0.25
TPMT activity (pmol/h/mg protein)	425 ± 20	295 ± 24	0.024
HPRT activity (nmol/h/mg protein)	621 ± 12	576 ± 57	1.0

### Micro-array analysis

PCA graphically discriminated between the 6-MP resistant and sensitive 11 cell lines. The first three components explained 37.6% of the total variance (Additional file 3: Figure S2).

Of the 23,786 genes analyzed in the micro-array, 943 and 210 had different basal expression levels in the two groups at *P* values of <0.01 and <0.001, respectively. Of the 210 genes significantly different at the *P* < 0.001 level, 84 were upregulated and 126 downregulated in the resistant compared to the sensitive cell lines (Additional file 1: Table S4).

#### Transcriptomic signature

**Figure 2:**
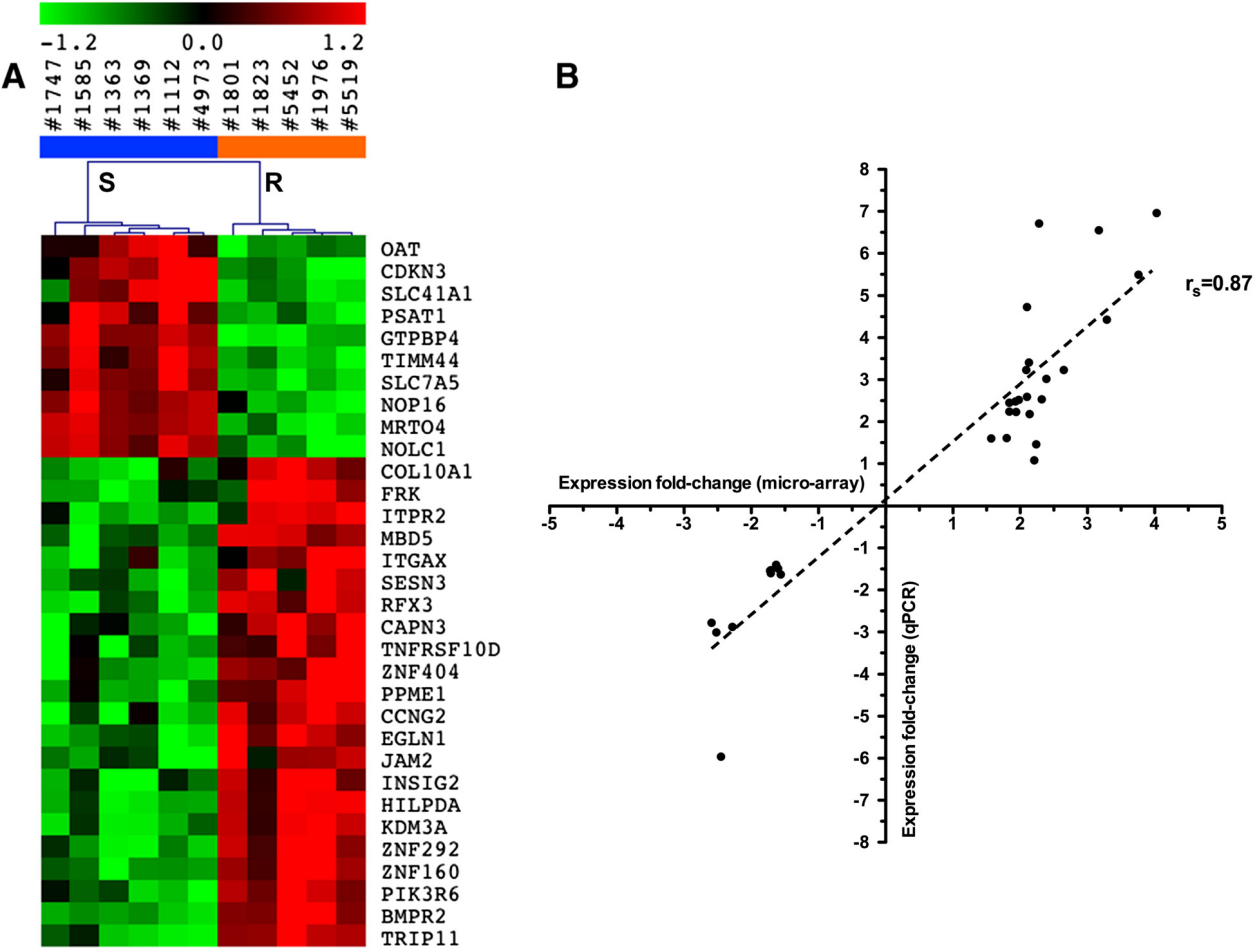
Transcriptomic signature characterizing cell lines resistant to thiopurines. **(A)** Heatmap of the transcriptomic signature validated by qPCR for resistant (orange) compared to sensitive (blue) cell lines. Overexpressed genes are in red and underexpressed genes in green. Fold-changes in the relative expression of each gene are reported in Additional file 1: Table S5. **(B)** Validation by qPCR of the transcriptomic signature including 32 genes. Fold-changes in the relative expression of each of the 32 genes as determined using qPCR (X axis) and micro-array (Y axis), with *GUSB* as the reference gene (*r*_*s*_ = 0.87; *P* <0.0001).

For identifying the most relevant genes that were differentially expressed in 6-MP resistant compared to sensitive cell lines, we performed PAM. This shrunken centroid algorithm identified 40 genomic loci that sorted the cell lines between resistant and sensitive, with a cross-validation error rate of 0.09 (Additional file 1: Table S5). To validate the expression signature determined from the micro-array, we performed RT-qPCR. Among the 40 genomic loci, seven were related to unknown transcripts, non-coding RNA, or unknown proteins and were consequently not selected for RT-qPCR. Furthermore, we were not able to amplify one gene (*CNR1*). The remaining 32 genes were validated for the transcriptomic signature using *GUSB* as the reference gene (*r*_*s*_ = 0.87; *P* <0.0001) (Figure 2). These results were reproducible using the three other reference genes, G*APDH*, *RPL13A*, and *B2M* (data not shown).

#### Comprehensive pathway analysis

**Figure 3:**
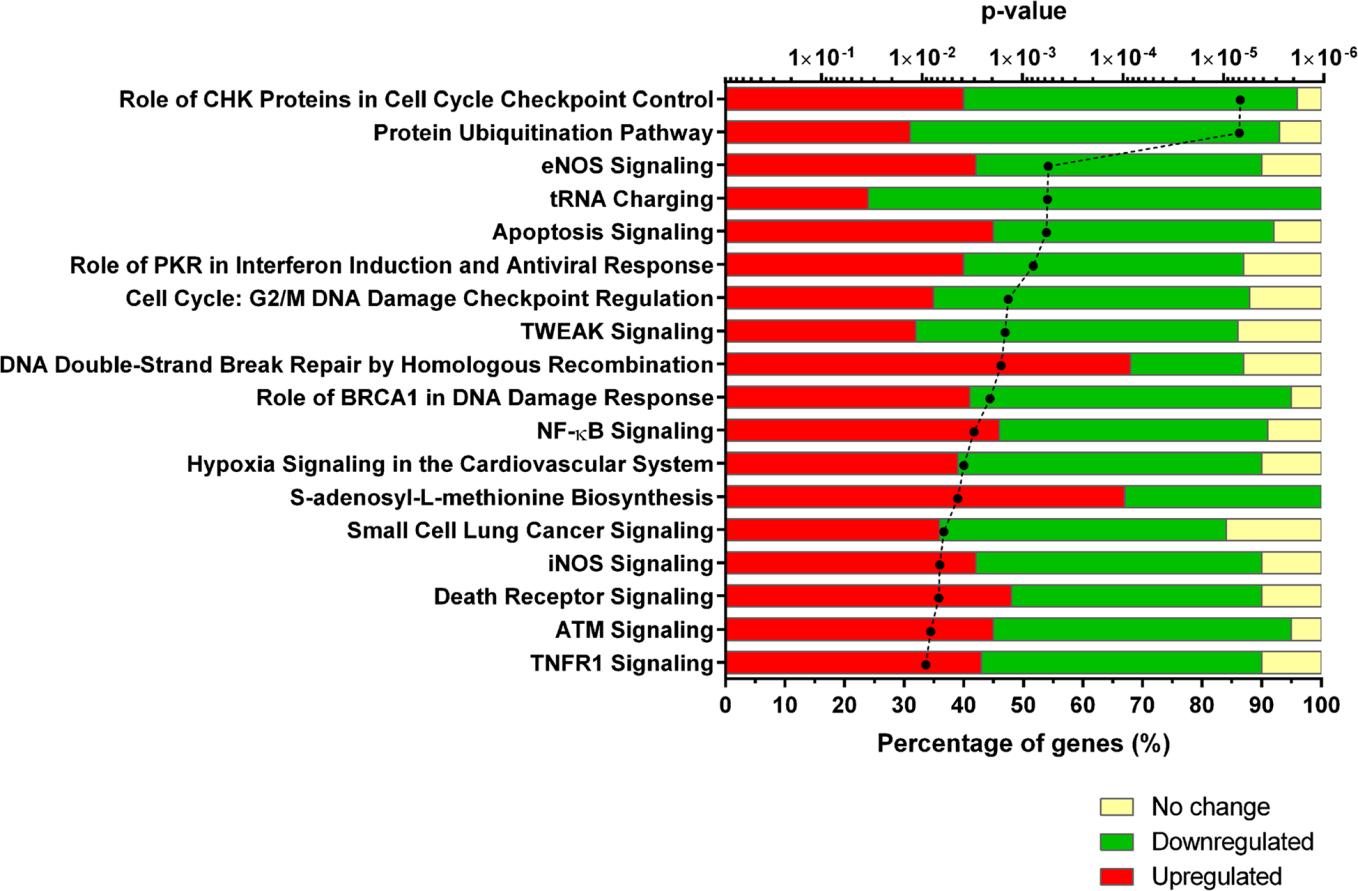
Top Ingenuity® canonical pathways enriched by genes that were significantly differentially expressed in resistant cell lines. The Ingenuity® canonical pathway analysis associates the 943 gene dataset with the canonical pathways in Ingenuity’s Knowledge Base and returns two measures of association: (1) a ratio of the number of genes from the list that maps to the pathway divided by the total number of genes that map to the same pathway, and (2) a *P* value of the Fisher’s exact test for each pathway. Ingenuity® canonical pathways associated with a *P* value <0.01 are presented.

The analysis of the GO biological processes involved in differential gene expression (*P* <0.01) led to 119 terms each containing at least five genes. After a Benjamini correction adjusted at a significance level (0.01/119), we found 10 GO terms, including 122 single genes, involved in the phenotype difference (Table 2; Additional file 1: Table S6). Half these terms were involved in cell proliferation functions and the other half chiefly in RNA processes. Ingenuity® predicted that nine upstream transcriptional regulators were activated (*TP53*, *CD24*, *NUPR1*, *CDKN1A*) or inhibited (*FOXM1*, *FLI1*, *MYC*, *CSF2*, *CCDN1*) in resistant cell lines (*P* <0.01). These regulators targeted 128 of the differentially expressed genes (Table 3). Furthermore 18 canonical pathways played a significant biological role in thiopurine resistance (*P* <0.01) (Figure 3, Additional file 1: Table S7 and Table S8).

**Table 2:** **Gene ontology terms involved in the phenotypic difference between resistant and sensitive cell lines**

**GO ref**	**GO term**	**Genes (n)**	**Fold enrichment**	***P*** **value**	**Benjamini corrected** ***P*** **value**	
GO:0022613	Ribonucleoprotein complex biogenesis	27	3.53	4.17E-08	9.26E-05	RNA processes
GO:0006396	RNA processing	52	2.24	9.74E-08	1.08E-04	
GO:0034660	ncRNA metabolic process	29	2.97	4.99E-07	3.69E-04	
GO:0042254	Ribosome biogenesis	20	3.86	8.66E-07	4.81E-04	
GO:0034470	ncRNA processing	23	2.89	1.44E-05	3.99E-03	
GO:0022402	Cell cycle process	49	2.04	3.45E-06	1.53E-03	Cell cycle
GO:0007049	Cell cycle	60	1.82	8.61E-06	3.18E-03	
GO:0000279	M phase	33	2.36	1.08E-05	3.41E-03	
GO:0022403	Cell cycle phase	38	2.16	1.54E-05	3.79E-03	
GO:0000278	Mitotic cell cycle	35	2.23	1.91E-05	4.24E-03	

Significance level Benjamini corrected *P* value <0.01.

List of genes represented by these terms is presented in Additional file 1: Table S6.

**Table 3:** **Upstream regulator analysis of the resistant compared to the sensitive cell lines**

**Symbol**	**Description**	**Regulator type**	**Predicted activation state**	***P*** **value of overlap (<0.01)**	**Target genes differentially expressed in micro-array**
FOXM1	Forkhead box M1	Transcription regulator	Inhibited	4.71E-07	ATF2, BIRC5, BUB1B, CCNA2, CCNB1, CDC25A, CDKN3, CENPA, CENPB, FOXM1, GTSE1, MMP2, PLK1
TP53	Tumor protein p53	Transcription regulator	Activated	6.41E-05	ACLY, ACTA2, APAF1, ATG10, BIRC5, BTG1, BUB1B, CCNA2, CCNB1, CCNG2, CDC25A, CDC25C, CDKN3, CHUK, CLPP, CYB5A, DDB2, E2F1, EDA2R, EIF4G3, FASN, FBXW7, GNL3, GTSE1, HBEGF, HIF1A, HK2, IPO7, JMJD1C, KIF23, KPNA2, MET, MMP2, NDC80, NPEPPS, NUP153, OAT, ORAI2, PDK1, PIDD, PLK1, PSMD12, PSME3, PVT1, RAD50, RAD54B, RBL2, RFC3, RPS6KB1, SCO2, SFPQ, SGPL1, SLC19A1, SPC25, SQLE, STARD4, TIMM44, TLR6, TMEM97, TRIM28, UBE2C, USO1, USP14, ZFP36L1
FLI1	Fli-1 proto-oncogene, ETS transcription factor	Transcription regulator	Inhibited	6.18E-04	DDX21, NIP7, NOL6, NOLC1, SNRPB, TCP1
MYC	v-myc avian myelocytomatosis viral oncogene homolog	Transcription regulator	Inhibited	7.33E-04	ASNS, BIRC2, BUB1B, CCNA2, CCNB1, CCNG2, CDC25A, CNBP, DCTPP1, DDB2, DKC1, E2F1, FASN, FOXM1, FTH1, GOT1, GTF2B, HIF1A, HK2, IPO7, ITGA6, MAT2A, NOLC1, OAT, PDK1, PHF21A, PLK1, SHMT2, SLC1A5, SLC3A2, SLC7A5, SNRPD1, SPRR2G, TIMM23, TMEM126A, TXNRD1, UBE2C
CD24	CD24 molecule	Other	Activated	8.97E-04	CHAC1, DNAJC13, JMJD1C, MBNL1, RAD50, SCAF11, SFPQ, SPG11, USO1, VPS13B, VPS13C
NUPR1	Nuclear protein, transcriptional regulator, 1	Transcription regulator	Activated	2.14E-03	BTG1, BUB1B, CCNA2, CDC25C, CDCA2, CDCA8, CHUK, EGLN1, FUT11, GINS1, GPCPD1, GTSE1, HBEGF, HILPDA, HIST1H2AB/HIST1H2AE, HIST1H3A, HK2, KDM3A, KIF23, MAT2A, MTFMT, MTFR2, PDK1, PLK1, RAB7L1, RIMKLA, RNU11, SPC25, UBIAD1, ZFP36L1, ZNF259
CDKN1A	Cyclin-dependent kinase inhibitor 1A (p21, Cip1)	Kinase	Activated	2.36E-03	ACTA2, BIRC5, CCNA2, CCNB1, CDC25A, CDC25C, FOXM1, PLK1, RBL2
CSF2	Colony stimulating factor 2 (granulocyte-macrophage)	Cytokine	Inhibited	4.34E-03	BIRC5, BUB1B, CCNA2, CDC123, CDCA2, CDCA8, CEACAM1, FOXM1, ITGAX, MAT2A, PLK1, PPIF, SKA1, SLC1A5, SPC25, TRIP13, UBE2C
CCND1	Cyclin D1	Other	Inhibited	5.45E-03	BIRC5, BRWD1, CCNA2, CDCA2, CDCA8, CENPN, E2F1, FOXM1, MTFR2, PLK1, SPC25, STARD4, TBCK, TOR3A, TRIP13

Ingenuity® Pathway Analysis was used to determine the most relevant upstream regulators, according to target gene expressions in the micro-array. Changes are expressed in resistant cell lines, using sensitive cell lines as reference.

The *P* value of overlap was used to rank the significance associated for each upstream regulator. The *P* value indicates the significance of the overlap between the genes targeted by the upstream regulator in the database and the data from micro-arrays, without taking into account the regulation direction. The activation state makes predictions about potential regulators by using information about the direction of gene regulation and can be used to infer the activation state of a putative regulator. Results with a *P* value <0.01 are presented in this table.

## Discussion

Using a pharmacogenomic LCL-based model, we performed a comprehensive analysis of molecular resistance to thiopurines. To our knowledge, we identified for the first time a 32-gene transcriptomic signature predicting thiopurine resistance. Of the 32 genes, 22 were upregulated and 10 downregulated. Our transcriptomic analysis of untreated and phenotypically selected cell lines identified potential biomarkers for thiopurine resistance and suggested metabolic pathways that might constitute therapeutic targets for overcoming thiopurine resistance.

### Model validity

Our model based on LCL growth inhibition is of interest for studying thiopurine resistance, as thiopurines are used to target lymphoblasts in patients with ALL. Moreover, in autoimmune diseases, in which thiopurine therapy also plays a major role, lymphocytes are the target cells and lymphocyte apoptosis must be achieved to induce immunosuppression [37]. Growth inhibition by 6-MP was closely correlated to growth inhibition by azathioprine and by 6-TG. A proof-of-concept study based on a panel of LCLs showed high goodness-of-fit values for linear regression plots comparing growth inhibition profiles of paired drugs acting via a shared pathway [25]. Thus, cell lines resistant to 6-MP were also resistant to azathioprine and to 6-TG, indicating that our results on 6-MP resistance are likely to be representative of thiopurine drugs. In addition, LCLs from patients with Lesch-Nyhan syndrome, which lacked HPRT activity because of a recessive X-linked gene defect, were completely resistant to thiopurines. This finding reflects the inability of HPRT-deficient cells to bioactivate thiopurines into active cytotoxic metabolites, mainly 6-thioguanine nucleotides [11,13]. The resistance phenotype of HPRT-deficient LCLs constitutes an appropriate positive control for our *in vitro* model. Altogether, these findings support the validity of our LCL-based pharmacogenomics model for studying thiopurine resistance.

### Targeted analysis of thiopurine metabolizing enzymes

The antiproliferative effects of the purine analogue 6-MP are due to metabolites that have three mechanisms of action: inhibition of *de novo* purine synthesis; cell cycle arrest due to metabolite incorporation into DNA; and, particularly in lymphocytes, increased apoptosis due to Rac1 inhibition [8]. We first studied variations in the main thiopurine-metabolizing enzymes known to influence the pharmacological response to thiopurine drugs. We did not study xanthine oxidase, which is not present in lymphocytes. HPRT activity was not significantly different between resistant and sensitive cell lines. Variations in HPRT activity have been reported to be associated with thiopurine resistance and hematological toxicity [13,38]. However, HPRT activity does not vary widely within the general population and probably has little influence on clinical outcomes [39]. By contrast, the considerable interindividual variations in TPMT activity in the general population, which are related to genetic polymorphisms, affect both the toxicity and the efficacy of thiopurine agents [8]. Higher ALL remission rates have been reported in patients with a decreased TPMT activity [7]. Interestingly, TPMT activity was slightly higher in the resistant than in the sensitive cell lines in our study. This finding confirms the association between drug metabolism and thiopurine susceptibility found in our LCLs, in keeping with data from treated patients [7]. However, *TPMT* expression levels were not significantly different between 6-MP resistant and sensitive LCLs, probably because *TPMT* regulation is mainly post-translational [40,41].

### Comprehensive pathway analysis

Transcriptomic analysis identified 210 genes that were significantly upregulated or downregulated in resistant cell lines. Our GO analysis of these genes showed that 10 GO terms were enriched in these genes, including five related with the cell cycle, most notably the M phase. Ingenuity® pathway analysis predicted *CDKN1A* activation in resistant cell lines. *CDKN1A* encodes a potent cyclin-dependent kinase (CDK) inhibitor, also called p21^WAF1/CIP1^, which binds to and inhibits the cyclin-CDK2 or -CDK4 complexes, preventing the phosphorylation of critical CDK substrates and blocking cell cycle progression [42]. Thus, *CDKN1A* acts as a negative regulator of cell cycle progression at G1. More specifically, when located in the nucleus, p21^WAF1/CIP1^ controls the cell cycle and DNA replication, whereas cytoplasmic p21^WAF1/CIP1^ has been implicated in apoptosis inhibition [42]. A study of human cancer cells showed that increased p21^WAF1/CIP1^ levels, related to phosphatidylinositol 3-kinase (PI3K) pathway inhibition, induced chemoresistance by causing a cell cycle delay [43]. Moreover, resistance to another anticancer drug, taxol, has been reported in breast-cancer cells exhibiting upregulation of p21^WAF1/CIP1^ [44]. A recent study also identified p21^WAF1/CIP1^ expression as a major factor in resistance to promising anticancer drugs acting within the cell cycle [45]. The tumor-suppressor protein p53 tightly controls p21^WAF1/CIP1^, through which it mediates the p53-dependent cell cycle G1-phase arrest in response to a variety of stress stimuli. We found upregulation of p53 target genes and of ATM, a p53 upstream regulator, which is activated in response to DNA damage, to be related with thiopurine resistance. Moreover, p21^WAF1/CIP1^ mediates NUPR1-induced chemoresistance, and our analysis predicted NUPR1 activation in resistant cell lines [46].

Another p53 target gene is *TNFRSF10D*, which encodes tumor necrosis factor-related apoptosis-inducing ligand receptor 4 (TRAILR4) and whose overexpression was a component of the transcriptomic signature identified in our study. Increased *TNFRSF10D* expression has been found to be associated with chemoresistance [47]. Taken in concert, these results suggest upregulation of the ATM/p53/p21 DNA damage response pathway in resistant cell lines, with resulting inhibition of the cyclin-CDK2 or -CDK4 complexes and cell cycle arrest (Additional file 4: Figure S3).

Furthermore, a study of the genome-scale protein-interaction profile of p53 showed that GTPBP4 was a p53 interactor involved in 60S ribosome biogenesis [48]. This nucleolar GTP-binding protein, whose downregulation was a component of our transcriptomic signature in resistant cell lines, has been reported to activate p53 when silenced [48]. Finally, in keeping with p53 activation, *MYC* inhibition, associated with cell cycle repression, was predicted in resistant cell lines [49].

Our canonical pathway analysis identified 18 significant processes that were differentially expressed between 6-MP resistant and sensitive cell lines, among which at least five were related to DNA repair in response to damage, including ‘Role of CHK proteins in cell cycle checkpoint control’ and ‘cell cycle: G2/M DNA damage checkpoint regulation’. Many anticancer drugs acting as antimetabolites require involvement of the DNA mismatch repair (MMR) system to exert their cellular responses [50]. The primary function of the MMR system is to edit and repair DNA replication errors and DNA damage [50]. Loss of MMR has been observed in a variety of human cancers and is associated with resistance to several anticancer agents such as etoposide, cisplatin, carboplatin, and 5-fluorouracil [50]. Human cancer cell lines lacking the MMR system were resistant to high doses of 6-TG compared to MMR-proficient cell lines [51]. A study established that cell cycle arrest in G2-M after thiopurine treatment was mediated by single-strand breaks in MMR-proficient cells [52]. In keeping with these findings, a recent study showed that high expression of PKCζ, a protein kinase believed to stabilize the MMR protein MSH2, increased the response to thiopurine therapy in pediatric patients with ALL [53]. A characteristic of resistant cell lines demonstrated by our transcriptomic analysis was a significantly decreased expression of *RFC3* and *POLDIP2*, both known to interact with the MMR system and cell replication. Thus, MMR deficiency and, possibly, the expression levels of *RFC3* and *POLDIP2*, may help to predict thiopurine resistance. Furthermore, a study based on MOLT-4 cell lines also suspected the role of alterations in the MMR system in the resistance phenotype to 6-MP [54]. Moreover, an increase in induced mutations after 6-TG treatment has been reported in MMR-deficient cell lines [51]. In a study of 228 children with ALL previously treated with anticancer agents including thiopurines, *CCNG2* under-expression was a risk factor for treatment-related myeloid leukemia (t-ML) [55]. CCNG2, a negative regulator of cell cycle progression independent from p53, is induced in cell cycle arrest in response to DNA damage [56]. Alterations in *CCNG2* expression may enhance cell cycle progression and contribute to failure of the cells to respond to DNA-damage stimuli that would otherwise promote exit from the cell cycle; subsequently, the proliferation of cells carrying misrepaired DNA may lead to leukemic transformation [55]. Conversely, our resistant cell lines exhibited a high level of *CCNG2* expression that might stop the cell cycle at the G1/S phase, preventing the MMR system from promoting thiopurine susceptibility and thereby contributing to thiopurine resistance.

Finally, we found under-expression of *ARHGDIA*, encoding for Rho-GDP dissociation inhibitor alpha (RhoGDIα) in resistant cell lines. RhoGDIα regulates and sequesters in cytoplasm inactive GDP-bound forms of RhoGTPase, including Rac1, a molecular target inhibited by thiopurine nucleotides [57]. Thus, RhoGDIα prevents RhoGTPase from being recruited at the cellular membrane where it can be activated. Decreased *ARHGDIA* expression may therefore increase the amount of potentially active Rac1, preventing effective Rac1 inhibition by the thiopurine nucleotide 6-thioguanosine triphosphate (6-TGTP). RhoGDIα thinly regulates RhoGTPase activation, involved in cellular processes and contributing to tumor invasion and metastasis [57,58]. Moreover, loss of RhoGDIα has been previously associated with tamoxifene resistance [59]. This mechanism, related to thiopurine pharmacodynamics, may contribute to thiopurine resistance by reducing 6-TGTP induced apoptosis *via* Rac1 inhibition in lymphocytes [8]. It may represent an original biomarker of thiopurine resistance.

### Study limitations

The transcriptomic content of LCLs includes many genes from diverse cellular pathways and has proven valuable for studying genome-wide individual differences in alternative mRNA splicing [60]. The reliability of the association between genomic analysis results and drug response phenotypes in LCL-based models deserves discussion. *In vitro* biological noise may limit the usefulness of LCLs as a pharmacogenomics research tool [61]. Several parameters measured in our study, including EBV and mtDNA copy numbers, intracellular ATP level, and basal cell growth rate, have been described as potential confounding factors influencing the LCL drug response phenotype [20,25,61,62]. These non-genetic variables unrelated to the genomic status of the cell line can alter growth inhibition. They are of major concern when using LCLs produced from different EBV strains and generated by different laboratories [25]. In our study, however, all the LCLs came from a single biobank (NLGIP) and were generated by a single lab using the same stock of B-95 EBV-expressing marmoset cell line [25]. Furthermore, our resistant and sensitive cell lines exhibited no significant differences regarding the EBV and mtDNA copy numbers or intracellular ATP levels measured during growth inhibition experiments. These parameters are thus unlikely to have affected our transcriptomic analysis. However, we observed a trend toward a lower basal cell growth rate in thiopurine-resistant compared to thiopurine-sensitive cell lines. Thus, thiopurine susceptibility may be, at least in part, associated with the basal cell growth rate. This finding has been previously observed in a mechanistic mathematical modelling of 6-MP resistance [54]. As discussed above, some of the 210 genes identified in our study may be related to the cell growth rate and cell cycle. When Elion and Hitchings synthesized 6-MP as a drug for treating ALL, their goal was to selectively inhibit nucleic acid synthesis in rapidly dividing cells such as leukemic cells [63]. Thus, our study unveils molecular pathways associated with the mechanisms of action of thiopurines such as cell cycle arrest upon incorporation of thioguanine nucleotides.

## Conclusion

**Figure 4:**
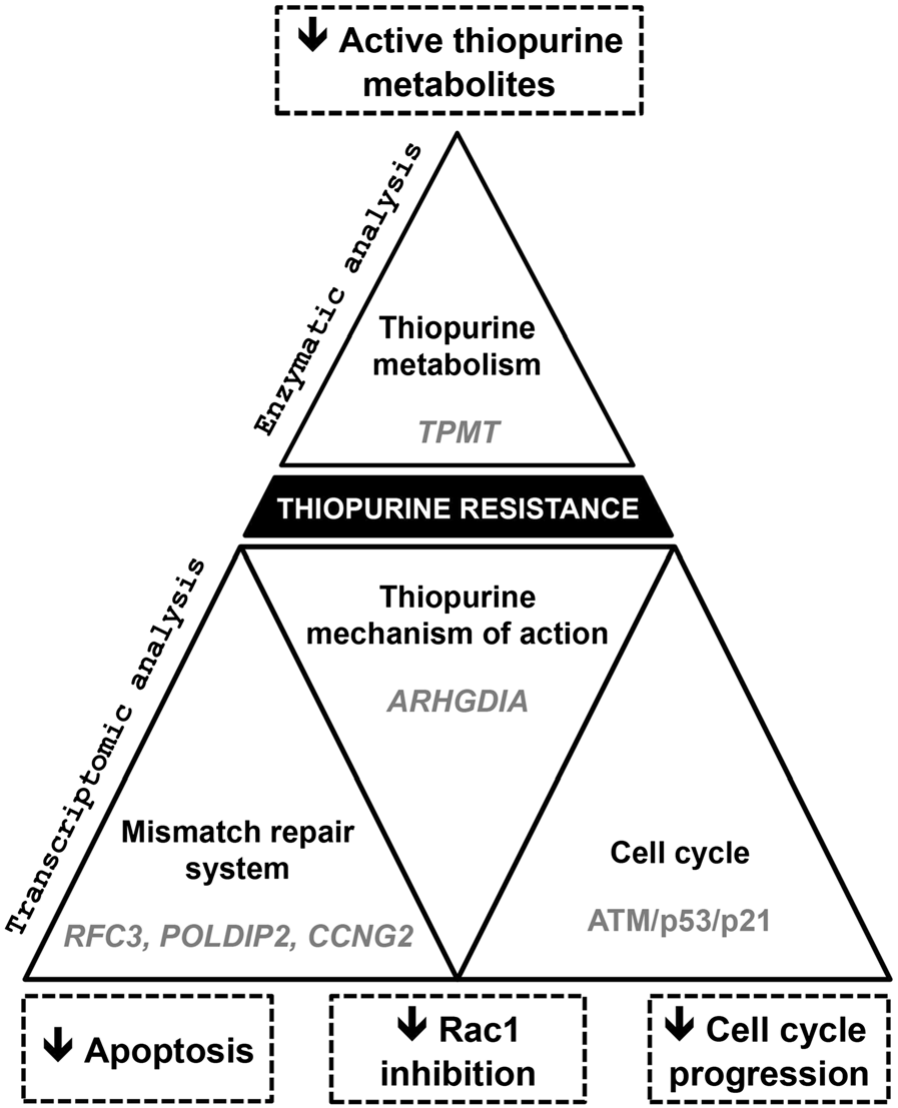
Molecular insight into thiopurine resistance. Proposal mechanisms and candidate biomarkers contributing to thiopurine cellular resistance phenotype in lymphoblastoid cell lines.

In conclusion, our study using an LCL-based model identified a transcriptomic signature of thiopurine resistance. We used a well-established pharmacogenomics approach involving transcriptomic profiling of basal mRNA in a cell-line model, taking advantage of the extra power afforded by analyzing extreme phenotype cell lines [64,65]. Thus, genome-wide transcriptomic analysis of LCLs coupled with drug susceptibility phenotyping can identify novel candidate genes and pathways that may help to explain individual response to thiopurine drugs (Figure 4). Our study provides new insights into the molecular mechanisms underlying thiopurine resistance suggesting potential research focus for developing tailored medicine.

## Additional files

Additional file 1:
**Data S1.** Methodology of microfluidic-based RT-qPCR assay. **Table S2.** Primers used for the microfluidic-based quantitative RT-qPCR assays. **Table S3.** Primers used for the copy number variation assays of EBV and mitochondrial DNA in lymphoblastoid cell lines. **Table S4.** List of the 210 genes significantly different at the *P* <0.001 level in the resistant compared to the sensitive cell lines. **Table S5.** List of the 40 genomic loci identified using a PAM on micro-array data. **Table S6.** List of genes involved in gene ontology terms. **Table S7.** Top Ingenuity® canonical pathways enriched by genes that were significantly differentially expressed in resistant cell lines. **Table S8.** Genes involved in the top Ingenuity® canonical pathways. Additional file 2: Figure S1. Correlations between growth inhibitions by thiopurine drugs among 53 lymphoblastoid cell lines. **(A)** Correlation between growth inhibition by 6-MP (2 μM) and by azathioprine (5 μM) (*r*_*spearman*_ = 0.95; *P* <0.0001). **(B)** Correlation between growth inhibition by 6-MP (2 μM) and by 6-TG (0.5 μM) (*r*_*spearman*_ = 0.81; *P* <0.0001). Data of growth inhibition by 6-TG (0.5 μM) are available only for 43 cell lines. Additional file 3: Figure S2. Principal component analysis for the 11 lymphoblastoid cell lines used for the transcriptomic analysis. #xxxx, cell line ID; R, resistant cell line; S, sensitive cell line. Additional file 4: Figure S3. Functional downstream *CDKN1A* network.

## Abbreviations

6-MP: 6-mercaptopurine
6-TG: 6-thioguanine
6-TGTP: 6-thioguanosine triphosphate
ALL: Acute lymphoblastic leukemia
AMP: Adenosine monophosphate
ATP: Adenosine triphosphate
cDNA: Complementary DNA
DAVID: Database for annotation visualization and integrated discovery
EBV: Epstein-Barr virus
gDNA: genomic DNA
GEO: Gene expression omnibus
GO: Gene ontology
HPRT: Hypoxanthine-guanine phosphoribosyltransferase
IMP: Inosine monophosphate
LCL: Lymphoblastoid cell lines
mtDNA: mitochondrial DNA
NCBI: National center for biotechnology information
NLGIP: National laboratory for the genetics of Israeli populations
PAM: Prediction analysis of microarrays
PCA: Principal component analysis
RMA: Robust multi-array average
RT-qPCR: Reverse transcriptase-quantitative polymerase chain reaction
TPMT: Thiopurine *S*-methyltransferase
