# Supplementary material for: Molecular insight into thiopurine resistance: transcriptomic signature in lymphoblastoid cell lines
Source: Genome Med. 2015 Apr 18;7(1):37. doi: 10.1186/s13073-015-0150-6 (PMC4443628; doi:10.1186/s13073-015-0150-6)
Supplement: Additional file 1: — Data S1. Methodology of microfluidic-based RT-qPCR assay. Table S2. Primers used for the microfluidic-based quantitative RT-qPCR assays. Table S3. Primers used for the copy number variation assays of EBV and mitochondrial DNA in lymphoblastoid cell lines. Table S4. List of the 210 genes significantly different at the P <0.001 level in the resistant compared to the sensitive cell lines. Table S5. List of the 40 genomic loci identified using a PAM on micro-array data. Table S6. List of genes involved in gene ontology terms. Table S7. Top Ingenuity® canonical pathways enriched by genes that were significantly differentially expressed in resistant cell lines. Table S8. Genes involved in the top Ingenuity® canonical pathways. [file 13073_2015_150_MOESM1_ESM.docx]

**SUPPLEMENTARY DATA & TABLES**

**Data S1. Methodology of microfluidic-based RT-qPCR assay**

*cDNA synthesis*

Reverse transcription (RT) was performed using 2 μg of RNA and High Capacity cDNA Reverse Transcription kit (Applied Biosystems [CA, USA]). cDNA were then diluted to a concentration equivalent to 10 ng/μL of RNA.

*Primer design*

For each gene, a first set of two primers was chosen in consecutive exons spanning a large intron. Exon sequences for primer design were extracted from Ensembl (GRCh37). When possible, one of the two primers was designed directly on the exon-exon junction to prevent possible persistent gDNA or unspliced intron amplification bias. These pairs were used in the pre-amplification step. When this primer design was not possible directly on the exon-exon junction, a third primer (a forward junction primer) compatible with the reverse primer of the first two was designed manually to encompass the junction. This pair was used in the second PCR step, *i.e*. qPCR on Biomark^®^.

All primers were designed using Primer3 (<http://frodo.wi.mit.edu/primer3/>) and checked for specificity by *in silico* PCR (<http://genome.ucsc.edu/cgi-bin/hgPcr>) on genome assembly and genes. Detailed primer sequences can be found above in Table S2.

*qPCR*

qPCR was performed on the BioMark™ (Fluidigm, The Netherlands) in a microfluidic multiplex 96x96 dynamic array chip according to the Fluidigm Advanced Development Protocol with EvaGreen^®^ (PN100-1208B1). One chip was used to quantify all the 44 transcripts. Briefly, a 14-cycle pre-amplification reaction was performed for each sample in 10µL by pooling all primer pairs (final concentration, 50 nM), 4.5 µL cDNA, and 5 µL 2X TaqMan^®^ PreAmp Master Mix (Life Technologies) according to manufacturer conditions. Then, for each individual assay, 5 µL 10X Assay Mix containing 9 µM forward primer, 9 µM reverse primer and 1X Assay Loading Reagent was loaded into one of the Assay inlets on the chip. The following solution (5 µL) was loaded in Sample inlets: 1.25 µL pre-amplified sample previously diluted to 1:5 in low TE Buffer, 2.5 µL 2X TaqMan^®^ Gene Expression Master Mix (Life Technologies), 0.25 µL 20X DNA Binding Dye Sample Loading Reagent (Fluidigm, PN 100-0388), 0.25 µL 20X EvaGreen^®^ (Biotium) and 0.75 µL low TE Buffer. The BioMark's default cycling program with a final melting was used to amplify fragments. All experiments were done in four replicates.

*RT-qPCR analysis*

Cycle threshold (Ct) values were obtained using BioMark™ Gene Expression Data Analysis version 3.0.2, according to Fluidigm’s recommendations for EvaGreen Gene Expression. Relative gene expression of resistant cell lines compared to sensitive ones was analyzed using the 2^-ΔΔCt^ method with *GUSB* as reference gene as recommended for transcriptomic analysis of LCLs (Livak et Schmittgen 2001; de Brouwer, van Bokhoven, et Kremer 2006): *ΔΔCt**= Ct_target_gene_ - Ct_GUSB_)_”resistant”_LCLs_ - (Ct_target_gene_ - Ct_GUSB_)_”sensitive”_LCLs_*. Results were then confirmed on the 3 others reference genes (*GAPDH*/*RPL13A*/*B2M*).

| **Transcript** | **Symbol** | **Forward Primer** | **Reverse Primer** | **Forward Junction Primer** | **Amplicon size (bp)** |
| --- | --- | --- | --- | --- | --- |
| ENST00000374580 | BMPR2 | CAGCACTGCGGCTGCTTC | CGGATCTTTAAACGCACATAGCC | - | 55 |
| ENST00000397163 | CAPN3 | GTCTGGAAGAGACCTCCGGAAA | TCAGTTCTGTTGGCTCCATCAAT | - | 68 |
| ENST00000316355 | CCNG2 | CTCCGGCACGATGAAGGATT | AGACGTTCAACAACCCGAGAAGT | - | 80 |
| ENST00000335183 | CDKN3 | GGGAGATCTTGTCTTGTAGCTGCTT | CTGTCTATGGCTTGCTCTGGTGA | - | 77 |
| ENST00000243222 | COL10A1 | GCTCTCCTCTTACTGCTATACCTTTACTCTTT | CACAGGCATAAAAGGCCCACTAC | - | 92 |
| ENST00000606080 | FRK | AGCTGGGACCTGGATCTTTAAGC | CGAATTTGTGAGCCACTACACCA | - | 85 |
| ENST00000360803 | GTPBP4 | CGTCCGCCAAGGACTTCATAG | AACGGTTGGAGTCTTTCGTTGAG | - | 62 |
| ENST00000268296 | ITGAX | CTCCTCCTGTTCACAGCCTTAGC | CAGCTCCTCTGTGTCCAAGTTGA | - | 60 |
| ENST00000381340 | ITPR2 | CACCTTGAAAAGGCAGTCTCTGA | TTAGTGGATGACAGATGTGTGGTG | - | 87 |
| ENST00000312912 | KDM3A | ACAGTGGCCTGCAATAACGTACA | GATCTCCCAGAAAGCGAACAGAA | - | 83 |
| ENST00000330263 | MRTO4 | CAGCGCGATGCCCAAAT | GCAGTTTTGGTTAAGGAGACTTTCTTG | - | 54 |
| ENST00000405356 | NOLC1 | AAAGCGACAGGAGCTACACAGC | GTCTAAGAGGGAAGAGGCATTGG | - | 51 |
| ENST00000510123 | NOP16 | CCATGGCCTTCACCTTTCTCTTA | ACGCTAAATCGGTACGGCAGAA | - | 96 |
| ENST00000311434 | PIK3R6 | CTCCACATGCCTTGGTTGCTC | ACCTCCAGAGGAGCGTGCAG | - | 82 |
| ENST00000398427 | PPME1 | CTTCCCTCTCGCCCACCTCTAC | GCTTTCTTCCAGGGCCCATT | - | 79 |
| ENST00000376588 | PSAT1 | GGCATTAGTGTTCTTGAAATGAGTCAC | CAATTCCCGCACAAGATTCTCT | - | 84 |
| ENST00000382004 | RFX3 | TCTGTACCTGCTGTACCTGTTGTTG | CTAGTCAAGCAGCAGTGCCTACG | - | 72 |
| ENST00000536441 | SESN3 | AGAATCCACAATCCCAAAGTTGC | TCTGCGTTTGTGATCTTGCTAATG | - | 80 |
| ENST00000261622 | SLC7A5 | GATGGGCAGGGAGATGATGA | TGATCAACCCCTACAGAAACCTG | - | 53 |
| ENST00000270538 | TIMM44 | TCTGACTCGATGGTTTTGTATTTCC | ACGAGGCCAGAAGGCTAGAAGA | - | 73 |
| ENST00000267622 | TRIP11 | ATTTTCGGCATCAGACAGTGCT | ATTCTTGCCCAGAGTGCATCAGT | - | 69 |
| ENST00000429604 | ZNF160 | TCAAAATGACACAGTCCCAGAGAA | TACAGGGACGTGATGTTGGAGAA | - | 62 |
| ENST00000369577 | ZNF292 | CAGCTGTGCCAGACACTCCTAGA | TGGATAGCCACTGTGTATACCTCCA | - | 92 |
| ENST00000366641 | EGLN1 | TACCTCCACTTACCTTGGCATCC | TCCAAATGGAGATGGAAGATGTG | - | 77 |
| ENST00000367137 | SLC41A1 | GTGGCCTGCACCTGGATG | AACATTGGACACATGGACACACC | - | 80 |
| ENST00000535130 | CNR1 | GGTATCTGCAAGGCCATCTAGGA | GGCAGAGTTGCGTTTGGAAATA | GAGAAAGTGACCCACAGGGGGCAATCCCGA | 73 |
| ENST00000257696 | HILPDA | CGCTGGTGCTTAGTAACCGACTT | ACAGAGCTGCCTTCTCCTTCTGA | ATCCACTCCCGGCTGTTCCCCCGGAGGGTC | 63 |
| ENST00000245787 | INSIG2 | GTGGAGGAGGAAGAGGCGGTA | CAAAGACTGACGCTTCAACGGTA | GGTCCCAGAAGATGGCGGAGGCGGGGGATT | 85 |
| ENST00000480456 | JAM2 | GACCCTCGACCTCCTCAGAGC | CTTTTGGGGCAGAAAACCCATAG | GCGCTACCTGGTGGTCGCCCTGGGCTATCA | 59 |
| ENST00000407073 | MBD5 | ACCAGCCACCCGTCAGAGAG | ACTTTTGAGTGCCTTCCCTGGAG | CCACCCGTCAGAGAGGGACATGCGCACTCC | 49 |
| ENST00000368845 | OAT | CATCGTAACTGGTTGGGTCTGTG | CGTAAGTGGGGCTATACCGTGAA | CAACGTCCTACCCCAGAAGTTCCCAGCTGC | 87 |
| ENST00000312584 | TNFRSF10D | ACTGTCTGCTGGGGAACTTCGT | GGACCCCAAGATCCTTAAGTTCG | GGGATGGTGGCAGAGTCAACCCGGACCGGC | 78 |
| ENST00000587539 | ZNF404 | TTTCCATGTCTCCTGATGGTACG | CGGATCAGAGGGATTTGTACAGAGA | GTTGCTTTCAGTTGTGAAATTAAAGTCCAA | 89 |
| ENST00000304895 | GUSB | CTGCTGGCTACTACTTGAAGATG | GAGTTGCTCACAAAGGTCAC | - |  |
| ENST00000229239 | GAPDH | ATGGAAATCCCATCACCATC | GACTCCACGACGTACTCAGC | - |  |
| ENST00000391857 | RPL13A | CCTGGAGGAGAAGAGGAAAGAGA | GAGGACCTCTGTGTATTTGTCAA | - |  |
| ENST00000558401 | B2M | AGATGAGTATGCCTGCCGTG | GCGGCATCTTCAAACCTCCA | - |  |

**Table S2. Primers used for the microfluidic-based quantitative RT-qPCR assays**

| **Gene** | **Forward primer** | **Reverse primer** | **Probe** |
| --- | --- | --- | --- |
| EBV DNA polymerase | 5’-GACGATCTTGGCAATCTCT-3’ | 5’-TGGTCATGGATCTGCTAAACC-3’ | 5’6FAM-CCACCTCCACGTGGATCACGA-MGB/NFQ-3’ |
| mtDNA *ND2* | 5’-TGTTGGTTATACCCTTCCCGTACTA-3’ | 5’-CCTGCAAAGATGGTAGAGTAGATGA-3’ | 5’6FAM-CCCTGGCCCAACCC-MGBNFQ-3’ |
| *NRF1* | 5’-CTCGGTGTAAGTAGCCACAT-3’ | 5’-GAGTGACCCAAACCGAACAT-3’ | 5’VIC-CACTGCATGTGCTTCTATGGTAGCCA-MGB/NFQ-3’ |

**Table S3. Primers used for the copy number variation assays of EBV and mitochondrial DNA in lymphoblastoid cell lines**

| **Entrez Gene ID** | **Symbol** | **Description** | **Chromosome location** | **Fold-change** | **p-value** |
| --- | --- | --- | --- | --- | --- |
| 23560 | GTPBP4 | GTP binding protein 4 | 10p15-p14 | -1.56251 | 1.30E-06 |
| 6897 | TARS | threonyl-tRNA synthetase | 5p13.2 | -1.41513 | 3.94E-06 |
| 51154 | MRTO4 | mRNA turnover 4 homolog (S. cerevisiae) | 1p36.13 | -1.71154 | 6.42E-06 |
| 109 | ADCY3 | adenylate cyclase 3 | 2p23.3 | -1.49016 | 8.69E-06 |
| 2870 | GRK6 | G protein-coupled receptor kinase 6 | 5q35 | -1.32366 | 9.48E-06 |
| 65244 | SPATS2 | spermatogenesis associated, serine-rich 2 | 12q13.12 | 1.34969 | 1.69E-05 |
| 659 | BMPR2 | bone morphogenetic protein receptor, type II (serine/threonine kinase) | 2q33-q34 | 1.57367 | 1.87E-05 |
| 7884 | SLBP | stem-loop binding protein | 4p16.3 | -1.42207 | 2.08E-05 |
| 692075 | SNORD6 | small nucleolar RNA, C/D box 6 | 11q21 | -1.48847 | 2.59E-05 |
| 5991 | RFX3 | regulatory factor X, 3 (influences HLA class II expression) | 9p24.2 | 2.31724 | 2.60E-05 |
| 29923 | HILPDA | hypoxia inducible lipid droplet-associated | 7q32.1 | 4.03008 | 2.65E-05 |
| 9221 | NOLC1 | nucleolar and coiled-body phosphoprotein 1 | 10q24.32 | -1.5987 | 3.22E-05 |
| 396 | ARHGDIA | Rho GDP dissociation inhibitor (GDI) alpha | 17q25.3 | -1.41129 | 3.52E-05 |
| 3654 | IRAK1 | interleukin-1 receptor-associated kinase 1 | Xq28 | -1.43088 | 3.59E-05 |
| 8192 | CLPP | ClpP caseinolytic peptidase, ATP-dependent, proteolytic subunit homolog (E. coli) | 19p13.3 | -1.13659 | 3.81E-05 |
| 124808 | CCDC43 | coiled-coil domain containing 43 | 17q21.31 | -1.25137 | 4.08E-05 |
| 25956 | SEC31B | SEC31 homolog B (S. cerevisiae) | 10q24.31 | 1.45234 | 4.21E-05 |
| 55379 | LRRC59 | leucine rich repeat containing 59 | 17q21.33 | -1.36213 | 5.32E-05 |
| 1314 | COPA | coatomer protein complex, subunit alpha | 1q23.2 | 1.17063 | 5.85E-05 |
| 4126 | MANBA | mannosidase, beta A, lysosomal | 4q22-q25 | 1.54478 | 5.98E-05 |
| 54583 | EGLN1 | egl nine homolog 1 (C. elegans) | 1q42.1 | 1.9282 | 6.32E-05 |
| 10452 | TOMM40 | translocase of outer mitochondrial membrane 40 homolog (yeast) | 19q13 | -1.57103 | 6.71E-05 |
| 9321 | TRIP11 | thyroid hormone receptor interactor 11 | 14q31-q32 | 1.79961 | 7.20E-05 |
| 55818 | KDM3A | lysine (K)-specific demethylase 3A | 2p11.2 | 2.10125 | 7.35E-05 |
| 10469 | TIMM44 | translocase of inner mitochondrial membrane 44 homolog (yeast) | 19p13.3-p13.2 | -1.63001 | 7.52E-05 |
| 23036 | ZNF292 | zinc finger protein 292 | 6q14.3 | 1.83528 | 7.64E-05 |
| 51594 | NBAS | neuroblastoma amplified sequence | 2p24 | 1.23372 | 7.83E-05 |
| 26797 | SNORD52 | small nucleolar RNA, C/D box 52 | 6p21.33 | -1.50947 | 7.85E-05 |
| 23081 | KDM4C | lysine (K)-specific demethylase 4C | 9p24.1 | 1.55882 | 7.97E-05 |
| 57763 | ANKRA2 | ankyrin repeat, family A (RFXANK-like), 2 | 5q12-q13 | 1.56409 | 8.22E-05 |
| 8140 | SLC7A5 | solute carrier family 7 (amino acid transporter light chain, L system), member 5 | 16q24.3 | -2.5869 | 8.35E-05 |
| 5373 | PMM2 | phosphomannomutase 2 | 16p13 | -1.3172 | 8.48E-05 |
| 9188 | DDX21 | DEAD (Asp-Glu-Ala-Asp) box helicase 21 | 10q21 | -1.52386 | 8.59E-05 |
| 29914 | UBIAD1 | UbiA prenyltransferase domain containing 1 | 1p36.22 | -1.53522 | 9.09E-05 |
| 10212 | DDX39A | DEAD (Asp-Glu-Ala-Asp) box polypeptide 39A | 19p13.12 | -1.39447 | 9.11E-05 |
| 91369 | ANKRD40 | ankyrin repeat domain 40 | 17q21.33 | -1.58783 | 9.52E-05 |
| 26817 | SNORD34 | small nucleolar RNA, C/D box 34 | 19q13.3 | -1.62204 | 1.05E-04 |
| 5898 | RALA | v-ral simian leukemia viral oncogene homolog A (ras related) | 7p15-p13 | -1.26319 | 1.05E-04 |
| 8480 | RAE1 | RAE1 RNA export 1 homolog (S. pombe) | 20q13.31 | -1.23209 | 1.07E-04 |
| 100506046 | GAS5-AS1 | GAS5 antisense RNA 1 | - | -1.6346 | 1.12E-04 |
| 54881 | TEX10 | testis expressed 10 | 9q31.1 | -1.28101 | 1.12E-04 |
| 79171 | RBM42 | RNA binding motif protein 42 | 19q13.12 | -1.3629 | 1.18E-04 |
| 442902 | MIR330 | microRNA 330 | 19q13.32 | -1.39969 | 1.19E-04 |
| 6421 | SFPQ | splicing factor proline/glutamine-rich | 1p34.3 | -1.37054 | 1.21E-04 |
| 4144 | MAT2A | methionine adenosyltransferase II, alpha | 2p11.2 | -1.43314 | 1.29E-04 |
| 80208 | SPG11 | spastic paraplegia 11 (autosomal recessive) | 15q14 | 1.41767 | 1.31E-04 |
| 79691 | QTRTD1 | queuine tRNA-ribosyltransferase domain containing 1 | 3q13.31 | -1.25662 | 1.31E-04 |
| 2805 | GOT1 | glutamic-oxaloacetic transaminase 1, soluble (aspartate aminotransferase 1) | 10q24.1-q25.1 | -1.59304 | 1.32E-04 |
| 57035 | C1orf63 | chromosome 1 open reading frame 63 | 1p36.13-p35.1 | 1.54113 | 1.43E-04 |
| 4329 | ALDH6A1 | aldehyde dehydrogenase 6 family, member A1 | 14q24.3 | 1.50757 | 1.43E-04 |
| 1033 | CDKN3 | cyclin-dependent kinase inhibitor 3 | 14q22 | -1.71173 | 1.52E-04 |
| 1386 | ATF2 | activating transcription factor 2 | 2q32 | 1.35466 | 1.53E-04 |
| 79660 | PPP1R3B | protein phosphatase 1, regulatory subunit 3B | 8p23.1 | 1.55902 | 1.53E-04 |
| 54931 | TRMT10C | tRNA methyltransferase 10 homolog C (S. cerevisiae) | 3q12.3 | -1.38843 | 1.53E-04 |
| 10328 | EMC8 | ER membrane protein complex subunit 8 | 16q24 | -1.45002 | 1.56E-04 |
| 7290 | HIRA | histone cell cycle regulator | 22q11.21 | -1.51288 | 1.63E-04 |
| 84135 | UTP15 | UTP15, U3 small nucleolar ribonucleoprotein, homolog (S. cerevisiae) | 5q13.2 | -1.49583 | 1.64E-04 |
| 157680 | VPS13B | vacuolar protein sorting 13 homolog B (yeast) | 8q22.2 | 1.41682 | 1.64E-04 |
| 317760 | ADAM20P1 | ADAM metallopeptidase domain 20 pseudogene 1 | 14q24.2 | 1.75848 | 1.67E-04 |
| 90338 | ZNF160 | zinc finger protein 160 | 19q13.42 | 1.93682 | 1.70E-04 |
| 5893 | RAD52 | RAD52 homolog (S. cerevisiae) | 12p13-p12.2 | 1.59764 | 1.71E-04 |
| 55186 | SLC25A36 | solute carrier family 25 (pyrimidine nucleotide carrier ), member 36 | 3q23 | 1.53412 | 1.75E-04 |
| 283571 | PROX2 | prospero homeobox 2 | 14q24.3 | 1.39544 | 1.75E-04 |
| 11065 | UBE2C | ubiquitin-conjugating enzyme E2C | 20q13.12 | -1.34418 | 1.84E-04 |
| 26807 | SNORD43 | small nucleolar RNA, C/D box 43 | 22q13 | -1.62558 | 1.85E-04 |
| 83989 | FAM172A | family with sequence similarity 172, member A | 5q15 | 1.31948 | 1.86E-04 |
| 54832 | VPS13C | vacuolar protein sorting 13 homolog C (S. cerevisiae) | 15q22.2 | 1.51273 | 1.87E-04 |
| 22992 | KDM2A | lysine (K)-specific demethylase 2A | 11q13.2 | 1.24264 | 1.93E-04 |
| 29928 | TIMM22 | translocase of inner mitochondrial membrane 22 homolog (yeast) | 17p13 | -1.39211 | 1.93E-04 |
| 546 | ATRX | alpha thalassemia/mental retardation syndrome X-linked | Xq21.1 | 1.67451 | 1.94E-04 |
| 50628 | GEMIN4 | gem (nuclear organelle) associated protein 4 | 17p13 | -1.27811 | 1.98E-04 |
| 472 | ATM | ataxia telangiectasia mutated | 11q22-q23 | 1.56433 | 2.02E-04 |
| 1058 | CENPA | centromere protein A | 2p23.3 | -1.41224 | 2.05E-04 |
| 4677 | NARS | asparaginyl-tRNA synthetase | 18q21.31 | -1.33695 | 2.15E-04 |
| 901 | CCNG2 | cyclin G2 | 4q21.1 | 2.14105 | 2.32E-04 |
| 10248 | POP7 | processing of precursor 7, ribonuclease P/MRP subunit (S. cerevisiae) | 7q22 | -1.29814 | 2.40E-04 |
| 10724 | MGEA5 | meningioma expressed antigen 5 (hyaluronidase) | 10q24.1-q24.3 | 1.39611 | 2.48E-04 |
| 6183 | MRPS12 | mitochondrial ribosomal protein S12 | 19q13.1-q13.2 | -1.31697 | 2.50E-04 |
| 83743 | GRWD1 | glutamate-rich WD repeat containing 1 | 19q13.33 | -1.47206 | 2.54E-04 |
| 51491 | NOP16 | NOP16 nucleolar protein | 5q35.2 | -1.72025 | 2.56E-04 |
| 9686 | VGLL4 | vestigial like 4 (Drosophila) | 3p25.3 | 1.60697 | 2.56E-04 |
| 49855 | SCAPER | S-phase cyclin A-associated protein in the ER | 15q24 | 1.46863 | 2.59E-04 |
| 55342 | STRBP | spermatid perinuclear RNA binding protein | 9q33.3 | 1.45223 | 2.65E-04 |
| 115817 | DHRS1 | dehydrogenase/reductase (SDR family) member 1 | 14q12 | 1.41899 | 2.69E-04 |
| 342908 | ZNF404 | zinc finger protein 404 | 19q13.31 | 2.38825 | 2.75E-04 |
| 10559 | SLC35A1 | solute carrier family 35 (CMP-sialic acid transporter), member A1 | 6q15 | 1.48095 | 2.79E-04 |
| 23082 | PPRC1 | peroxisome proliferator-activated receptor gamma, coactivator-related 1 | 10q24.32 | -1.52037 | 2.84E-04 |
| 1839 | HBEGF | heparin-binding EGF-like growth factor | 5q23 | -1.35451 | 2.85E-04 |
| 10142 | AKAP9 | A kinase (PRKA) anchor protein (yotiao) 9 | 7q21-q22 | 1.5168 | 2.91E-04 |
| 8772 | FADD | Fas (TNFRSF6)-associated via death domain | 11q13.3 | -1.26727 | 2.95E-04 |
| 5522 | PPP2R2C | protein phosphatase 2, regulatory subunit B, gamma | 4p16.1 | -1.35437 | 3.03E-04 |
| 100506948 | - | uncharacterized LOC100506948 | - | 1.86823 | 3.09E-04 |
| 90784 | - | uncharacterized LOC90784 | 2p11.2 | -1.57168 | 3.09E-04 |
| 84295 | PHF6 | PHD finger protein 6 | Xq26.3 | 1.26109 | 3.10E-04 |
| 22852 | ANKRD26 | ankyrin repeat domain 26 | 10p12.1 | 1.58926 | 3.12E-04 |
| 81929 | SEH1L | SEH1-like (S. cerevisiae) | 18p11.21 | -1.25825 | 3.13E-04 |
| 100506516 | - | uncharacterized LOC100506516 | - | 2.49955 | 3.19E-04 |
| 26792 | SNORD57 | small nucleolar RNA, C/D box 57 | 20p13 | -1.45516 | 3.28E-04 |
| 154743 | C7orf60 | chromosome 7 open reading frame 60 | 7q31.1 | 1.75585 | 3.30E-04 |
| 2495 | FTH1 | ferritin, heavy polypeptide 1 | 11q13 | -1.54533 | 3.35E-04 |
| 1643 | DDB2 | damage-specific DNA binding protein 2, 48kDa | 11p12-p11 | 1.5131 | 3.48E-04 |
| 25980 | AAR2 | AAR2 splicing factor homolog (S. cerevisiae) | 20pter-q12 | -1.35702 | 3.48E-04 |
| 9394 | HS6ST1 | heparan sulfate 6-O-sulfotransferase 1 | 2q21 | 1.46128 | 3.52E-04 |
| 64858 | DCLRE1B | DNA cross-link repair 1B | 1p13.2 | -1.34635 | 3.52E-04 |
| 8896 | BUD31 | BUD31 homolog (S. cerevisiae) | 7q22.1 | -1.2833 | 3.58E-04 |
| 1268 | CNR1 | cannabinoid receptor 1 (brain) | 6q14-q15 | 2.91315 | 3.60E-04 |
| 84549 | MAK16 | MAK16 homolog (S. cerevisiae) | 8p12 | -1.45128 | 3.61E-04 |
| 339975 | - | uncharacterized LOC339975 | 4q35.2 | 1.16838 | 3.63E-04 |
| 92935 | MARS2 | methionyl-tRNA synthetase 2, mitochondrial | 2q33.1 | -1.37001 | 3.65E-04 |
| 29107 | NXT1 | NTF2-like export factor 1 | 20p12-p11.2 | -1.48657 | 3.67E-04 |
| 10197 | PSME3 | proteasome (prosome, macropain) activator subunit 3 (PA28 gamma; Ki) | 17q21 | -1.51946 | 3.72E-04 |
| 6628 | SNRPB | small nuclear ribonucleoprotein polypeptides B and B1 | 20p13 | -1.27265 | 3.73E-04 |
| 3182 | HNRNPAB | heterogeneous nuclear ribonucleoprotein A/B | 5q35.3 | -1.386 | 3.78E-04 |
| 9648 | GCC2 | GRIP and coiled-coil domain containing 2 | 2q12.3 | 1.48356 | 3.80E-04 |
| 10921 | RNPS1 | RNA binding protein S1, serine-rich domain | 16p13.3 | -1.26917 | 3.81E-04 |
| 5347 | PLK1 | polo-like kinase 1 | 16p12.2 | -1.45289 | 3.86E-04 |
| 51400 | PPME1 | protein phosphatase methylesterase 1 | 11q13.4 | 1.98386 | 3.87E-04 |
| 25771 | TBC1D22A | TBC1 domain family, member 22A | 22q13.3 | 1.35259 | 3.92E-04 |
| 339175 | METTL2A | methyltransferase like 2A | 17q23.2 | -1.49058 | 3.94E-04 |
| 55269 | PSPC1 | paraspeckle component 1 | 13q12.11 | -1.26914 | 3.96E-04 |
| 55209 | SETD5 | SET domain containing 5 | 3p25.3 | 1.15909 | 3.97E-04 |
| 55893 | ZNF395 | zinc finger protein 395 | 8p21.1 | 1.68801 | 3.98E-04 |
| 139735 | ZFP92 | ZFP92 zinc finger protein | Xq28 | -1.29795 | 4.04E-04 |
| 55299 | BRIX1 | BRX1, biogenesis of ribosomes, homolog (S. cerevisiae) | 5p13.2 | -1.41137 | 4.04E-04 |
| 28741 | TRAJ14 | T cell receptor alpha joining 14 | 14q11 | 1.51584 | 4.18E-04 |
| 8565 | YARS | tyrosyl-tRNA synthetase | 1p35.1 | -1.35673 | 4.18E-04 |
| 6832 | SUPV3L1 | suppressor of var1, 3-like 1 (S. cerevisiae) | 10q22.1 | -1.24578 | 4.19E-04 |
| 10180 | RBM6 | RNA binding motif protein 6 | 3p21.3 | 1.30979 | 4.23E-04 |
| 7584 | ZNF35 | zinc finger protein 35 | 3p21.32 | -1.33252 | 4.23E-04 |
| 389118 | CDHR4 | cadherin-related family member 4 | 3p21.31 | 1.38177 | 4.23E-04 |
| 7296 | TXNRD1 | thioredoxin reductase 1 | 12q23-q24.1 | -1.60258 | 4.41E-04 |
| 6668 | SP2 | Sp2 transcription factor | 17q21.32 | -1.24147 | 4.48E-04 |
| 27332 | ZNF638 | zinc finger protein 638 | 2p13.1 | 1.51382 | 4.52E-04 |
| 55279 | ZNF654 | zinc finger protein 654 | 3p11.1 | 1.60706 | 4.57E-04 |
| 9136 | RRP9 | ribosomal RNA processing 9, small subunit (SSU) processome component, homolog (yeast) | 3p21.2 | -1.23422 | 4.59E-04 |
| 51426 | POLK | polymerase (DNA directed) kappa | 5q13 | 1.50196 | 4.59E-04 |
| 10024 | TROAP | trophinin associated protein | 12q13.12 | -1.35312 | 4.65E-04 |
| 93081 | TEX30 | testis expressed 30 | 13q33.1 | -1.66856 | 4.69E-04 |
| 9931 | HELZ | helicase with zinc finger | 17q24.2 | 1.60697 | 4.71E-04 |
| 317 | APAF1 | apoptotic peptidase activating factor 1 | 12q23 | 1.62627 | 4.84E-04 |
| 84948 | TIGD5 | tigger transposable element derived 5 | 8q24.3 | -1.46461 | 4.92E-04 |
| 55777 | MBD5 | methyl-CpG binding domain protein 5 | 2q23.1 | 2.21493 | 4.96E-04 |
| 101059976 | - | uncharacterized LOC101059976 | - | -1.21895 | 5.02E-04 |
| 619498 | SNORD74 | small nucleolar RNA, C/D box 74 | 1q25.1 | -1.64145 | 5.09E-04 |
| 8662 | EIF3B | eukaryotic translation initiation factor 3, subunit B | 7p22.3 | -1.25462 | 5.15E-04 |
| 9342 | SNAP29 | synaptosomal-associated protein, 29kDa | 22q11.21 | -1.30341 | 5.16E-04 |
| 29968 | PSAT1 | phosphoserine aminotransferase 1 | 9q21.2 | -2.51706 | 5.18E-04 |
| 123228 | SENP8 | SUMO/sentrin specific peptidase family member 8 | 15q23 | 1.587 | 5.24E-04 |
| 987 | LRBA | LPS-responsive vesicle trafficking, beach and anchor containing | 4q31.3 | 1.43056 | 5.25E-04 |
| 3709 | ITPR2 | inositol 1,4,5-trisphosphate receptor, type 2 | 12p11 | 2.64833 | 5.29E-04 |
| 54014 | BRWD1 | bromodomain and WD repeat domain containing 1 | 21q22.2 | 1.39546 | 5.50E-04 |
| 114599 | SNORD15B | small nucleolar RNA, C/D box 15B | 11q13.4 | -1.52378 | 5.52E-04 |
| 993 | CDC25A | cell division cycle 25A | 3p21 | -1.50737 | 5.59E-04 |
| 23451 | SF3B1 | splicing factor 3b, subunit 1, 155kDa | 2q33.1 | 1.15535 | 5.66E-04 |
| 118491 | TTC18 | tetratricopeptide repeat domain 18 | 10q22.2 | 1.41437 | 5.68E-04 |
| 51701 | NLK | nemo-like kinase | 17q11.2 | 1.4522 | 5.78E-04 |
| 221545 | C6orf136 | chromosome 6 open reading frame 136 | 6p21.33 | -1.27869 | 5.83E-04 |
| 26821 | SNORA74A | small nucleolar RNA, H/ACA box 74A | 5q31.2 | -1.53337 | 5.84E-04 |
| 9373 | PLAA | phospholipase A2-activating protein | 9p21 | -1.34371 | 5.85E-04 |
| 56652 | C10orf2 | chromosome 10 open reading frame 2 | 10q24 | -1.35143 | 5.99E-04 |
| 51602 | NOP58 | NOP58 ribonucleoprotein | 2q33.1 | -1.32113 | 6.01E-04 |
| 10607 | TBL3 | transducin (beta)-like 3 | 16p13.3 | -1.29781 | 6.13E-04 |
| 55277 | FGGY | FGGY carbohydrate kinase domain containing | 1p32.1 | 1.53705 | 6.22E-04 |
| 89849 | ATG16L2 | autophagy related 16-like 2 (S. cerevisiae) | 11q13.4 | 1.38052 | 6.27E-04 |
| 55177 | RMDN3 | regulator of microtubule dynamics 3 | 15q15.1 | -1.21533 | 6.30E-04 |
| 55142 | HAUS2 | HAUS augmin-like complex, subunit 2 | 15q15.2 | -1.44116 | 6.33E-04 |
| 79954 | NOL10 | nucleolar protein 10 | 2p25.1 | -1.29968 | 6.47E-04 |
| 146850 | PIK3R6 | phosphoinositide-3-kinase, regulatory subunit 6 | 17p13.1 | 1.84064 | 6.52E-04 |
| 79693 | YRDC | yrdC domain containing (E. coli) | 1p34.3 | -1.5035 | 6.53E-04 |
| 619571 | SNORD96A | small nucleolar RNA, C/D box 96A | 5q35.3 | -1.40851 | 6.83E-04 |
| 5983 | RFC3 | replication factor C (activator 1) 3, 38kDa | 13q13.2 | -1.34327 | 6.92E-04 |
| 9088 | PKMYT1 | protein kinase, membrane associated tyrosine/threonine 1 | 16p13.3 | -1.38381 | 6.93E-04 |
| 51204 | TACO1 | translational activator of mitochondrially encoded cytochrome c oxidase I | 17q23.3 | -1.35127 | 6.96E-04 |
| 56681 | SAR1A | SAR1 homolog A (S. cerevisiae) | 10q22.1 | -1.28402 | 7.05E-04 |
| 6079 | SNORD15A | small nucleolar RNA, C/D box 15A | 11q13.4 | -1.37549 | 7.17E-04 |
| 9097 | USP14 | ubiquitin specific peptidase 14 (tRNA-guanine transglycosylase) | 18p11.32 | -1.23244 | 7.27E-04 |
| 26808 | SNORD42B | small nucleolar RNA, C/D box 42B | 17q11 | -1.44515 | 7.45E-04 |
| 2706 | GJB2 | gap junction protein, beta 2, 26kDa | 13q11-q12 | -1.51575 | 7.48E-04 |
| 26793 | SNORD56 | small nucleolar RNA, C/D box 56 | 20p13 | -1.42307 | 7.62E-04 |
| 54812 | AFTPH | aftiphilin | 2p14 | 1.17972 | 7.69E-04 |
| 2645 | GCK | glucokinase (hexokinase 4) | 7p15.3-p15.1 | -1.3191 | 7.74E-04 |
| 79077 | DCTPP1 | dCTP pyrophosphatase 1 | 16p11.2 | -1.62844 | 7.77E-04 |
| 10105 | PPIF | peptidylprolyl isomerase F | 10q22-q23 | -1.83902 | 7.86E-04 |
| 51639 | - | splicing factor 3B, 14 kDa subunit | 2pter-p25.1 | -1.25629 | 7.93E-04 |
| 29078 | NDUFAF4 | NADH dehydrogenase (ubiquinone) complex I, assembly factor 4 | 6q16.1 | -1.59942 | 7.97E-04 |
| 2617 | GARS | glycyl-tRNA synthetase | 7p15 | -1.5618 | 8.02E-04 |
| 23469 | PHF3 | PHD finger protein 3 | 6q12 | 1.42343 | 8.13E-04 |
| 64785 | GINS3 | GINS complex subunit 3 (Psf3 homolog) | 16q21 | -1.3939 | 8.40E-04 |
| 6341 | SCO1 | SCO1 cytochrome c oxidase assembly protein | 17p13.1 | -1.27742 | 8.41E-04 |
| 54865 | GPATCH4 | G patch domain containing 4 | 1q22 | -1.44142 | 8.43E-04 |
| 54882 | ANKHD1 | ankyrin repeat and KH domain containing 1 | 5q31.3 | -1.36633 | 8.45E-04 |
| 825 | CAPN3 | calpain 3, (p94) | 15q15.1 | 2.13011 | 8.47E-04 |
| 178 | AGL | amylo-alpha-1, 6-glucosidase, 4-alpha-glucanotransferase | 1p21 | 1.28614 | 8.48E-04 |
| 6629 | SNRPB2 | small nuclear ribonucleoprotein polypeptide B | 20p12.1 | -1.25732 | 8.58E-04 |
| 51141 | INSIG2 | insulin induced gene 2 | 2q14.2 | 2.09098 | 8.59E-04 |
| 10623 | POLR3C | polymerase (RNA) III (DNA directed) polypeptide C (62kD) | 1q21.1 | -1.33482 | 8.64E-04 |
| 80209 | PROSER1 | proline and serine rich 1 | 13q13.3 | -1.31281 | 8.68E-04 |
| 6018 | RLF | rearranged L-myc fusion | 1p32 | 1.703 | 8.86E-04 |
| 5537 | PPP6C | protein phosphatase 6, catalytic subunit | 9q33.3 | -1.24986 | 9.14E-04 |
| 80205 | CHD9 | chromodomain helicase DNA binding protein 9 | 16q12.2 | 1.39803 | 9.17E-04 |
| 100130301 | - | uncharacterized LOC100130301 | 8q21.11 | 1.91133 | 9.21E-04 |
| 619208 | FAM229B | family with sequence similarity 229, member B | 6q21 | 1.39734 | 9.31E-04 |
| 56851 | EMC7 | ER membrane protein complex subunit 7 | 15q14 | -1.42295 | 9.43E-04 |
| 2595 | GANC | glucosidase, alpha; neutral C | 15q15.2 | 1.31685 | 9.49E-04 |
| 9779 | TBC1D5 | TBC1 domain family, member 5 | 3p24.3 | 1.46407 | 9.58E-04 |
| 23505 | TMEM131 | transmembrane protein 131 | 2q11.2 | 1.72794 | 9.66E-04 |
| 64149 | C17orf75 | chromosome 17 open reading frame 75 | 17q11.2 | -1.2821 | 9.83E-04 |
| 23585 | TMEM50A | transmembrane protein 50A | 1p36.11 | -1.18913 | 9.89E-04 |
| 114049 | WBSCR22 | Williams Beuren syndrome chromosome region 22 | - | -1.16534 | 9.89E-04 |
| 51124 | IER3IP1 | immediate early response 3 interacting protein 1 | 18q12 | -1.33271 | 9.89E-04 |

**Table S4. List of the 210 genes significantly different at the *P*<0.001 level in the resistant compared to the sensitive cell lines**

84 genes are upregulated and 126 are downregulated.

| **Entrez Gene ID** | **Symbol** | **Description** | **Chromosome location** | **Fold-change (micro-array)** | **p-value** | **Fold-change (qPCR)** |
| --- | --- | --- | --- | --- | --- | --- |
| 29923 | HILPDA | hypoxia inducible lipid droplet-associated | 7q32.1 | 4.03 | 2.65E-05 | 6.96 |
| 143686 | SESN3 | sestrin 3 | 11q21 | 3.76 | 1.04E-03 | 5.49 |
| 2444 | FRK | fyn-related kinase | 6q21-q22.3 | 3.29 | 3.07E-03 | 4.43 |
| 3687 | ITGAX | integrin, alpha X (complement component 3 receptor 4 subunit) | 16p11.2 | 3.17 | 2.77E-03 | 6.55 |
| 1268 | CNR1 | cannabinoid receptor 1 (brain) | 6q14-q15 | 2.91 | 3.60E-04 | - |
| 3709 | ITPR2 | inositol 1,4,5-trisphosphate receptor, type 2 | 12p11 | 2.65 | 5.29E-04 | 3.23 |
| 100506516 | LINC01176 | long intergenic non-protein coding RNA 1176 | 7p14.3 | 2.50 | 3.19E-04 | - |
| 342908 | ZNF404 | zinc finger protein 404 | 19q13.31 | 2.39 | 2.75E-04 | 3.02 |
| 5991 | RFX3 | regulatory factor X, 3 (influences HLA class II expression) | 9p24.2 | 2.32 | 2.60E-05 | 2.53 |
| 58494 | JAM2 | junctional adhesion molecule 2 | 21q21.2 | 2.28 | 1.97E-03 | 6.71 |
| 1300 | COL10A1 | collagen, type X, alpha 1 | 6q21-q22 | 2.24 | 1.75E-03 | 1.46 |
| 55777 | MBD5 | methyl-CpG binding domain protein 5 | 2q23.1 | 2.21 | 4.96E-04 | 1.08 |
| 901 | CCNG2 | cyclin G2 | 4q21.1 | 2.14 | 2.32E-04 | 2.18 |
| 825 | CAPN3 | calpain 3 (p94) | 15q15.1 | 2.13 | 8.47E-04 | 3.41 |
| 8793 | TNFRSF10D | tumor necrosis factor receptor superfamily, member 10d | 8p21 | 2.10 | 1.60E-03 | 4.73 |
| 55818 | KDM3A | lysine (K)-specific demethylase 3A | 2p11.2 | 2.10 | 7.35E-05 | 2.59 |
| 51141 | INSIG2 | insulin induced gene 2 | 2q14.2 | 2.09 | 8.59E-04 | 3.23 |
| 51400 | PPME1 | protein phosphatase methylesterase 1 | 11q13.4 | 1.98 | 3.87E-04 | 2.52 |
| 751816 | DSERG1 | Down syndrome encephalopathy related protein 1 | 9p24.2 | 1.97 | 1.05E-03 | - |
| 90338 | ZNF160 | zinc finger protein 160 | 19q13.42 | 1.94 | 1.70E-04 | 2.23 |
| 54583 | EGLN1 | egl nine homolog 1 (C. elegans) | 1q42.1 | 1.93 | 6.32E-05 | 2.48 |
| 100130301 | LOC100130301 | uncharacterized locus | 8q13.3 | 1.91 | 9.21E-04 | - |
| 100506948 | LOC100506948 | uncharacterized locus | 15q11.2 | 1.87 | 3.09E-04 | - |
| 146850 | PIK3R6 | phosphoinositide-3-kinase, regulatory subunit 6 | 17p13.1 | 1.84 | 6.52E-04 | 2.45 |
| 23036 | ZNF292 | zinc finger protein 292 | 6q14.3 | 1.84 | 7.64E-05 | 2.24 |
| 9321 | TRIP11 | thyroid hormone receptor interactor 11 | 14q31-q32 | 1.80 | 7.20E-05 | 1.61 |
| 317760 | ADAM20P1 | ADAM metallopeptidase domain 20 pseudogene 1 | 14q24.2 | 1.76 | 1.67E-04 | - |
| 154743 | C7orf60 | chromosome 7 open reading frame 60 | 7q31.1 | 1.76 | 3.30E-04 | - |
| 659 | BMPR2 | bone morphogenetic protein receptor II (serine/threonine kinase) | 2q33-q34 | 1.57 | 1.87E-05 | 1.60 |
| 23560 | GTPBP4 | GTP binding protein 4 | 10p15-p14 | -1.56 | 1.30E-06 | -1.63 |
| 9221 | NOLC1 | nucleolar and coiled-body phosphoprotein 1 | 10q24.32 | -1.60 | 3.22E-05 | -1.49 |
| 10469 | TIMM44 | translocase of inner mitochondrial membrane 44 homolog (yeast) | 19p13.3-p13.2 | -1.63 | 7.52E-05 | -1.40 |
| 100506046 | GAS5-AS1 | GAS5 antisense RNA 1 | 1q25.1 | -1.63 | 1.12E-04 | - |
| 51154 | MRTO4 | mRNA turnover 4 homolog (S. cerevisiae) | 1p36.13 | -1.71 | 6.42E-06 | -1.60 |
| 1033 | CDKN3 | cyclin-dependent kinase inhibitor 3 | 14q22 | -1.71 | 1.52E-04 | -1.53 |
| 51491 | NOP16 | NOP16 nucleolar protein | 5q35.2 | -1.72 | 2.56E-04 | -1.54 |
| 254428 | SLC41A1 | solute carrier family 41 (magnesium transporter), member 1 | 1q32.1 | -2.28 | 1.67E-03 | -2.88 |
| 4942 | OAT | ornithine aminotransferase | 10q26 | -2.45 | 2.15E-03 | -5.96 |
| 29968 | PSAT1 | phosphoserine aminotransferase 1 | 9q21.2 | -2.52 | 5.18E-04 | -3.01 |
| 8140 | SLC7A5 | solute carrier family 7 (amino acid transporter light chain, L system), member 5 | 16q24.3 | -2.59 | 8.35E-05 | -2.78 |

**Table S5. List of the 40 genomic loci identified using a PAM on micro-array data**

* dark grey boxes: no known transcript, non-coding RNA or unknown protein and thus not eligible for RT-qPCR

** light grey boxes: gene not validated by RT-qPCR

| **Biological functions** | **List of genes** |
| --- | --- |
| RNA processes  (n=122) | AARS, ADCY3, ARAP1, ATM, BCAS2, BCCIP, BIRC5, BRIX1, BUB1B, BYSL, CCNA2, CCNB1, CCNG2, CDC123, CDC25A, CDC25C, CDCA2, CDCA8, CDK5RAP1, CDKN3, CENPA, CENPV, DBR1, DCAF13, DDX20, DKC1, DMTF1, DST, E2F1, ESCO1, EXOSC9, FOXM1, GARS, GEMIN4, GSG2, GTPBP4, GTSE1, HAUS2, HCFC1, HNRNPF, ING4, INTS5, IVNS1ABP, KIAA1009, KIF23, KPNA2, LSM5, MAPRE1, MARS, MBNL1, MKI67IP, MPHOSPH8, MRPL41, MRTO4, NAE1, NARS, NDC80, NIP7, NOLC1, NOP56, NOP58, PFDN1, PINX1, PKMYT1, PLK1, PNPT1, POP4, POP7, PPIL3, PPP6C, PRMT5, PSMA4, PSMD12, PSMD6, PSME3, PUF60, PUS1, PUS10, QTRTD1, RACGAP1P, RAD1, RAD50, RAD52, RAD54B, RBL2, RBM39, RBM6, RNGTT, RNPS1, RPP30, RRP8, RRP9, RRS1, SDAD1, SEH1L, SESN3, SF1, SF3B1, SF3B14, SFPQ, SKA1, SLBP, SNRPB, SNRPB2, SNRPD1, SPC25, SYNCRIP, TARS, TBL3, THOC2, TRIP13, TRMT2B, TRMT6, U2AF1, UBE2C, UBR2, USP39, UTP11L, UTP15, YARS, ZFC3H1, ZNF638 |
| Cell cyle  (n=82) | AARS, ADCY3, ARAP1, ATM, BCCIP, BIRC5, BUB1B, CCNA2, CCNB1, CCNG2, CDC123, CDC25A, CDC25C, CDCA2, CDCA8, CDK5RAP1, CDKN3, CENPA, CENPV, DCAF13, DKC1, DMTF1, DST, E2F1, ESCO1, EXOSC9, FOXM1, GEMIN4, GSG2, GTSE1, HAUS2, HCFC1, ING4, INTS5, KIAA1009, KIF23, KPNA2, MAPRE1, MPHOSPH8, MRPL41, NAE1, NDC80, NOLC1, NOP56, NOP58, PFDN1, PINX1, PKMYT1, PLK1, POP4, POP7, PPP6C, PSMA4, PSMD12, PSMD6, PSME3, PUS1, PUS10, QTRTD1, RACGAP1P, RAD1, RAD50, RAD52, RAD54B, RBL2, RPP30, RRP8, RRP9, RRS1, SEH1L, SESN3, SF1, SKA1, SPC25, TBL3, TRIP13, TRMT2B, TRMT6, UBE2C, UBR2, UTP11L, UTP15 |

**Table S6. List of genes involved in gene ontology terms**

| **Canonical Pathways** | **p-value** | **Upregulated genes** | | **Downregulated genes** | | **No change** | |
| --- | --- | --- | --- | --- | --- | --- | --- |
| **Role of CHK Proteins in Cell Cycle Checkpoint Control** | 6.76E-06 | 23/57 | 40% | 32/57 | 56% | 2/57 | 4% |
| **Protein Ubiquitination Pathway** | 6.92E-06 | 82/264 | 31% | 164/264 | 62% | 18/264 | 7% |
| **eNOS Signaling** | 5.50E-04 | 57/136 | 42% | 65/136 | 48% | 14/136 | 10% |
| **tRNA Charging** | 5.62E-04 | 9/38 | 24% | 29/38 | 76% | 0/38 | 0% |
| **Apoptosis Signaling** | 5.75E-04 | 42/93 | 45% | 44/93 | 47% | 7/93 | 8% |
| **Role of PKR in Interferon Induction and Antiviral Response** | 7.76E-04 | 18/45 | 40% | 21/45 | 47% | 6/45 | 13% |
| **Cell Cycle: G2/M DNA Damage Checkpoint Regulation** | 1.38E-03 | 17/49 | 35% | 26/49 | 53% | 6/49 | 12% |
| **TWEAK Signaling** | 1.48E-03 | 12/37 | 32% | 20/37 | 54% | 5/37 | 14% |
| **DNA Double-Strand Break Repair by Homologous Recombination** | 1.62E-03 | 11/16 | 69% | 3/16 | 19% | 2/16 | 13% |
| **Role of BRCA1 in DNA Damage Response** | 2.09E-03 | 26/63 | 41% | 34/63 | 54% | 3/63 | 5% |
| **NF-κB Signaling** | 3.02E-03 | 83/180 | 46% | 81/180 | 45% | 16/180 | 9% |
| **Hypoxia Signaling in the Cardiovascular System** | 3.80E-03 | 26/67 | 39% | 34/67 | 51% | 7/67 | 10% |
| **S-adenosyl-L-methionine Biosynthesis** | 4.37E-03 | 2/3 | 67% | 1/3 | 33% | 0/3 | 0% |
| **Small Cell Lung Cancer Signaling** | 6.03E-03 | 31/85 | 36% | 40/85 | 47% | 14/85 | 16% |
| **iNOS Signaling** | 6.61E-03 | 20/48 | 42% | 23/48 | 48% | 5/48 | 10% |
| **Death Receptor Signaling** | 6.76E-03 | 30/63 | 48% | 27/63 | 43% | 6/63 | 10% |
| **ATM Signaling** | 8.13E-03 | 28/62 | 45% | 31/62 | 50% | 3/62 | 5% |
| **TNFR1 Signaling** | 9.12E-03 | 22/51 | 43% | 24/51 | 47% | 5/51 | 10% |

**Table S7. Top Ingenuity^®^ canonical pathways enriched by genes that were significantly differentially expressed in resistant cell lines**

Genes significantly regulated in resistant cell lines compared with sensitive cell lines. The Ingenuity^®^ canonical pathway analysis associates the 943 gene dataset with the canonical pathways in Ingenuity’s Knowledge Base and returns two measures of association: (i) a ratio of the number of genes from the list that maps to the pathway divided by the total number of genes that map to the same pathway, and (ii) a p-value of the Fisher’s exact test for each pathway. Ingenuity canonical pathways associated with a p-value < 0.01 are presented.

| **Canonical Pathways** | **List of genes** |
| --- | --- |
| **Role of CHK Proteins in Cell Cycle Checkpoint Control** | PPP2CA, PPP2R5B, PPP2R3B, E2F6, PPM1J, PPM1L, BRCA1, E2F2, ATM, CDC25A, TP53, E2F4, MDC1, RPA1, RFC1, RAD1, PPP2CB, PPP2R1A, PCNA, RFC4, PPP2R3A, PPP2R4, PPP2R2B, E2F1, ATMIN, TLK1, CLSPN, TLK2, CDK2, RFC3, PPP2R2A, MRE11A, E2F3, RAD50, CHEK1, RAD17, HUS1, E2F5, RFC2, SLC19A1, PPP2R5C, PPP2R2C, CHEK2, CDC25C, PPP2R5D, PLK1, RFC5, PPP2R5A, CDK1, NBN, RAD9A, CDKN1A, ATR, PPP2R5E, PPP2R1B |
| **Protein Ubiquitination Pathway** | CRYAB, USP45, USP20, DNAJC3, PAN2, DNAJC22, DNAJC16, UBE2Q1, DNAJC27, USP19, THOP1, USP6, SKP2, USP31, HSPA8, PSMB7, ZBTB12, PSMA5, PSMD12, PSMB1, PSMA4, ANAPC5, PSMD1, CRYAA, UBE2C, ANAPC2, USP18, PSMD7, HSPA6, HSPB8, DNAJC13, TAP1, USP39, HSPA1L, UBE4A, HSP90AB1, PSMC6, IFNG, DNAJB8, UCHL3, MED20, PSMD13, HSPA2, USP44, HLA-C, USP46, USP37, PSMA3, HSPA14, USP5, UBE3B, DNAJC15, HSPA5, TCEB1, USP8, HSPA4, SACS, UBE2B, SUGT1, NEDD4L, BIRC3, USP28, PSMB5, UBE4B, HSPB9, PSME2, PSMD6, DNAJC2, TCEB2, PSMD3, DNAJB14, DNAJC5, USP32, RBX1, USP29, BAP1, HSP90AA1, DNAJB6, USP51, VHL, HSPB1, UBE2I, B2M, HSPB3, USP21, USP14, CDC20, USP11, ANAPC10, USP54, DNAJC10, PSMD10, HSPA12B, DNAJC4, STUB1, DNAJB1, HSPB6, HSPA4L, UBB, UBE2R2, DNAJC1, HSPD1, UBE2D1, HSPA12A, NEDD4, HSCB, DNAJB11, PSMD2, UBE2E2, CDC34, PSMA7, DNAJB4, HSPB2, UBR2, PSMB8, USP48, DNAJC28, BAG1, USP10, UBE2D4, DNAJC30, ANAPC11, PSMC2, PSMA2, DNAJB12, USP9X, PSMD5, UBE2S, USP26, UBE2G2, UBD, TRAF6, ODF1, PSMB2, UBR1, HSPB11, DNAJC14, DNAJC5B, SMURF2, HSPB7, TAP2, USP24, PSMB10, HLA-A, PSMD9, CUL1, CDC23, DNAJA1, DNAJB13, SMURF1, USP3, UCHL1, USO1, USP27X, USP42, PSMD14, PARK2, AMFR, PSMB9, UBE2M, HLA-B, USP30, MDM2, USP33, PSMD8, DNAJC11, DNAJC24, PSME1, UBE2J1, CUL2, USP4, USP22, BTRC, USP49, USP25, BIRC2, FZR1, UBE2A, HSPA1A/HSPA1B, DNAJC6, UBE2D2, UBE2V2, FBXW7, DNAJB2, SKP1, PSMC5, USP7, USP53, DNAJC8, UCHL5, USP40, BRCA1, PSMA6, USP43, USP38, USP36, HSPA9, DNAJC19, PSMC4, BIRC6, DNAJC25, USP50, ANAPC4, PSMD11, UBE2L3, CBL, UBE2H, UBE2G1, HSPA13, DNAJC18, DNAJB7, UBE2E1, UBE2J2, PSMB3, DNAJC17, USP12, USP35, DNAJC12, USP2, ANAPC1, PSMB6, DNAJC5G, HSP90B1, USP13, USP47, USP16, UBE2V1, PSMB4, USP15, DNAJB3, HSPH1, PSMA1, USP1, DNAJB9, UBE3A, UBE2L6, XIAP, DNAJC21, UBA1, UBC, USP9Y, USP34, PSMC3, DNAJB5, UBE2D3, DNAJC7 |
| **eNOS Signaling** | KNG1, PRKAB1, PRKAB2, PIK3R1, ADCY4, CHRNB1, AQP8, NOS3, AQP4, VEGFA, NOSIP, CCNA1, PIK3CG, CAV1, CHRNA3, DNM2, AQP2, GUCY1B3, ATM, PIK3C2B, AQP5, AKT2, GUCY1A3, CHRNA10, ITPR1, CNGA3, HSPA8, AQP9, PLCG2, ITPR3, PIK3R6, PRKACA, PIK3CD, ESR2, PIK3CA, HSPA6, VEGFB, HSPA1L, BDKRB2, AQP3, AKT1, HSP90AB1, PIK3C3, CNGB3, PIK3R2, CASP8, CHRNA5, AQP6, CALML5, CNGA4, FLT4, GNAQ, PIK3C2G, ADCY6, PLCG1, LPAR3, HSPA2, NOSTRIN, CALM1, ADCY1, CHRNE, PRKACB, LPAR4, HSPA14, HSPA1A/HSPA1B, PIK3R4, HSPA5, HSPA4, CASP9, LPAR2, PROK1, ADCY5, PRKAA1, AQP7, CASP3, ITPR2, CHRNA9, HSPA9, AQP1, VEGFC, CNGA1, PIK3R3, ADCY9, PRKACG, GUCY1A2, HSP90AA1, ADCY10, ESR1, CAMK4, PIK3R5, CHRNB4, SLC7A1, CNGB1, PDGFC, PRKAG1, PGF, CCNA2, HSP90B1, STUB1, PRKAR1B, PRKAA2, AKT3, ADCY8, CNGA2, ADCY2, GNAS, CHRNA4, PIK3C2A, FLT1, ADCY3, PRKAR2A, LPAR6, PRKAR2B, PRKG1, LPAR1, LPAR5, PRKAG2, PIK3CB, KDR, BDKRB1, ADCY7, PRKAR1A |
| **tRNA Charging** | CARS, GARS, HARS, DARS2, QARS, NARS2, FARSB, NARS, PARS2, LARS, TARS2, FARS2, KARS, DARS, EARS2, SARS2, SARS, VARS, IARS, IARS2, RARS2, TARSL2, CARS2, MARS2, LARS2, TARS, HARS2, MARS, FARSA, EPRS, WARS2, WARS, YARS2, RARS, YARS, VARS2, AARS2, AARS |
| **Apoptosis Signaling** | MAP2K4, RAF1, MAPK1, BAD, HRAS, KRAS, DFFA, ACIN1, IKBKB, IKBKG, CASP9, MAPK3, MRAS, HTRA2, NFKBIB, BIRC3, FASLG, MCL1, CASP10, CAPN5, TP53, MAP3K14, CAPN6, PRKCQ, CASP3, RRAS, BIRC6, LMNA, BCL2L10, NFKB2, CAPN8, BCL2L1, CAPN1, CASP2, PLCG2, CAPN2, RPS6KA1, CAPN7, SPTAN1, BCL2A1, DIABLO, CYCS, TNF, CAPN3, GAS2, RELA, CAPN11, NFKBIE, MAP3K5, NFKB1, FAS, BCL2, CASP6, NFKBIA, PRKCE, BID, CHUK, CASP8, TNFRSF1B, MAP2K1, CAPN10, PRKCA, AIFM1, ENDOG, MAP2K7, NRAS, TNFRSF1A, APAF1, MAPK8, PLCG1, IKBKE, BAX, XIAP, BAK1, CDK1, PARP1, ROCK1, CASP12, RRAS2, CAPNS1, DFFB, CAPN9, MAP4K4, CASP7, BCL2L11, BIRC2 |
| **Role of PKR in Interferon Induction and Antiviral Response** | MAP2K6, RELA, TRAF3, NFKBIE, TAB2, IFNB1, EIF2S1, NFKB1, RNASEL, IKBKB, IKBKG, NFKBIA, CASP9, AKT1, MAP3K7, BID, TLR3, CHUK, TRAF5, CASP8, STAT1, NFKBIB, TP53, IFNG, CASP3, TNFRSF1A, APAF1, IKBKE, NFKB2, IRF1, ATF2, TRAF6, FADD, TRAF2, MAPK14, MAP2K3, EIF2AK2, CYCS, TNF |
| **Cell Cycle: G2/M DNA Damage Checkpoint Regulation** | CDKN2A, YWHAH, PTPMT1, CUL1, CCNB2, SKP1, PRKCZ, CHEK1, EP300, CDC25B, YWHAQ, TOP2B, KAT2B, GADD45A, TOP2A, BRCA1, CHEK2, ATM, TP53, PRKDC, CDC25C, YWHAG, YWHAE, CKS2, YWHAB, WEE1, CDK7, CCNB3, YWHAZ, RPRM, PLK1, MDM2, CDK1, SKP2, CCNB1, MDM4, CDKN1A, CKS1B, PKMYT1, BTRC, ATR, RPS6KA1, SFN |
| **TWEAK Signaling** | RELA, TRAF3, NFKBIE, NFKB1, TNFRSF12A, TRADD, CASP6, IKBKB, IKBKG, CASP9, NFKBIA, TNFSF12, BID, CHUK, CASP8, NFKBIB, BIRC3, TRAF1, MAP3K14, CASP3, APAF1, IKBKE, NFKB2, XIAP, BAG4, FADD, TRAF2, RIPK1, TNFRSF25, CYCS, CASP7, BIRC2 |
| **DNA Double-Strand Break Repair by Homologous Recombination** | LIG1, POLA1, ATRX, ABL1, MRE11A, RPA1, RAD50, NBN, RAD51, GEN1, RAD52, BRCA2, BRCA1, ATM |
| **Role of BRCA1 in DNA Damage Response** | FANCF, ATRIP, SMARCD2, UIMC1, RBL1, BRCC3, FANCB, RB1, E2F6, BRCA1, BRIP1, E2F2, ATM, TP53, E2F4, RBL2, FANCG, MDC1, RFC1, RPA1, FANCC, POU2F1, RFC4, SMARCA2, E2F1, C19orf40, FANCA, RFC3, FANCM, FAM175A, BARD1, RBBP8, MRE11A, E2F3, SMARCA4, MLH1, RAD50, CHEK1, FANCE, RAD51, FANCD2, GADD45A, E2F5, RFC2, SLC19A1, STAT1, BLM, CHEK2, C17orf70, ATF1, PLK1, RFC5, FANCL, NBN, MSH2, CDKN1A, MSH6, BRCA2, ATR, HLTF |
| **NF-κB Signaling** | RAF1, BMP4, TGFBR1, PIK3R1, TGFBR3, TAB2, HRAS, KRAS, BMPR1B, LCK, TLR10, PIK3CG, CSNK2A1, TDP2, TRAF5, NFKBIB, PDGFRB, ATM, PIK3C2B, AKT2, PRKCQ, RRAS, CREBBP, FGFR2, TBK1, NFKB2, TLR9, DDR1, TRAF6, ARAF, PLCG2, PIK3R6, PRKACA, PIK3CD, TNF, SIGIRR, MAP2K6, AZI2, PIK3CA, BMPR2, PRKCZ, EP300, TANK, TRADD, IL36G, AKT1, CARD10, NGFR, PIK3C3, NTRK1, IGF1R, TLR7, PDGFRA, TLR3, CSNK2B, PIK3R2, CASP8, TNFRSF1B, EGFR, TNFSF11, TNFRSF1A, MYD88, FLT4, PIK3C2G, MAPK8, IRAK3, GH1, IGF2R, IL36B, FADD, TRAF2, NTRK3, BCL10, LTA, PELI1, FCER1G, MAP3K8, BTRC, TIRAP, MAP3K3, IRAK4, PRKCB, PRKACB, TRAF3, CD40LG, TLR8, PIK3R4, TNFRSF17, TGFBR2, IL1R2, FGFR3, IKBKB, TNIP1, IKBKG, FGFR4, INS, TLR1, MRAS, LTBR, GSK3B, TNFSF13B, TAB1, TNFRSF11B, MAP3K14, HDAC2, FGFR1, TRGV9, IL36A, MALT1, IL37, TAB3, IL33, TLR2, PIK3R3, IL18, CD40, IL1RN, CARD11, IL36RN, ZAP70, PRKACG, FGFRL1, INSR, RELA, IL1A, BMP2, NFKBIE, PIK3R5, TNFAIP3, EGF, IL1F10, NGF, NFKB1, IRAK1, BRAF, NFKBIA, BMPR1A, MAP3K7, UBE2V1, AKT3, CHUK, MAP2K7, NRAS, PIK3C2A, FLT1, RELB, MAP3K1, HDAC1, IL1R1, TNFRSF11A, TLR4, CSNK2A2, NTRK2, GHR, RRAS2, RIPK1, TLR5, TLR6, TGFA, IL1B, PIK3CB, EIF2AK2, KDR, MAP4K4 |
| **Hypoxia Signaling in the Cardiovascular System** | UBE2A, UBE2D2, UBE2V2, NOS3, PTEN, ARNT, VEGFA, UBE2B, SUMO1, UBE2D4, ATF4, NFKBIB, ATM, TP53, P4HB, UBE2Q1, COPS5, CREB3, CREBBP, CSNK1D, BIRC6, UBE2S, CREB3L4, CREB5, SLC2A4, ATF2, UBE2G2, UBE2L3, UBE2H, UBE2G1, HIF1AN, HSP90AA1, UBE2E1, LDHA, VHL, UBE2I, UBE2C, UBE2J2, EPO, NFKBIE, HIF1A, EP300, HSP90B1, NFKBIA, AKT1, JUN, HSP90AB1, EDN1, CREB1, UBE2V1, UBE2M, NQO1, UBE2R2, MDM2, UBE2D1, UBE2L6, UBE2J1, UBE2E2, CDC34, UBE2D3 |
| **S-adenosyl-L-methionine Biosynthesis** | MAT1A, MAT2B, MAT2A |
| **Small Cell Lung Cancer Signaling** | TRAF3, SUV39H1, PIK3R1, PIK3R4, CCND1, PTEN, MYC, PTK2, IKBKB, RB1, IKBKG, CASP9, PIK3CG, TRAF4, TRAF5, NFKBIB, ATM, TP53, PIK3C2B, AKT2, CCNE2, TFDP1, CDK6, NFKB2, SKP2, TRAF6, PIK3R3, BCL2L1, MAX, E2F1, PIK3R6, PIK3CD, CYCS, RXRA, CDK2, NOS1, RELA, PIK3CA, PA2G4, NFKBIE, ABL1, CDK4, PIK3R5, NFKB1, CDKN2B, BCL2, NFKBIA, AKT1, PIK3C3, RARB, AKT3, BID, PIK3R2, CHUK, RXRB, TRAF1, FHIT, PIK3C2A, APAF1, PIK3C2G, IKBKE, SIN3A, PIAS3, RXRG, TRAF2, CCNE1, CKS1B, PIK3CB, PTGS2, CDKN1B, BIRC2 |
| **iNOS Signaling** | RELA, CAMK4, JAK1, MAPK1, NFKBIE, JAK2, MAPK13, NFKB1, MAPK11, IRAK1, IKBKB, IKBKG, NFKBIA, JUN, LBP, CHUK, NOS2, NFKBIB, STAT1, TAB1, IFNG, CALML5, MYD88, TYK2, CREBBP, IFNGR2, IKBKE, IFNGR1, HMGA1, NFKB2, IRAK3, MAPK12, IRF1, TRAF6, CALM1, TLR4, FOS, LY96, MAPK14, CD14, JAK3, IRAK4, IRAK2 |
| **Death Receptor Signaling** | MAP2K4, HSPB2, IKBKB, IKBKG, CASP9, CRADD, HTRA2, NFKBIB, BIRC3, FASLG, CASP10, TNFRSF21, MAP3K14, CASP3, TNFRSF10B, TBK1, NFKB2, DAXX, CASP2, TNFRSF25, HSPB7, CFLAR, CYCS, DIABLO, TNF, HSPB1, HSPB3, RELA, NFKBIE, TNFSF10, MAP3K5, NFKB1, FAS, BCL2, TANK, CASP6, TRADD, NFKBIA, TNFSF12, BID, TNFSF15, CHUK, CASP8, TNFRSF1B, TNFRSF10A, MAP2K7, TNFRSF1A, MAPK8, APAF1, IKBKE, XIAP, FADD, TRAF2, RIPK1, MAP4K4, CASP7, BIRC2 |
| **ATM Signaling** | MAP2K4, TP73, CCNB2, TDP1, MAPK13, CBX5, SMC1A, ATF4, BRCA1, CDC25A, ATM, TP53, SMC3, CREB3, CCNB3, CREBBP, MDC1, CREB3L4, CREB5, MAPK12, CCNB1, ATF2, MDM4, BRAT1, SMC2, H2AFX, SMC1B, MAPK10, TLK1, TLK2, CDK2, GADD45B, GADD45G, ABL1, MRE11A, MAPK11, RAD50, CHEK1, EP300, RAD51, NFKBIA, JUN, FANCD2, GADD45A, CREB1, BID, BLM, CHEK2, CDC25C, TRIM28, MAPK8, MAPK9, MDM2, CDK1, NBN, MAPK14, RAD9A, CDKN1A, TP53BP1 |
| **TNFR1 Signaling** | MAP2K4, RELA, NFKBIE, TNFAIP3, CDC42, NFKB1, TANK, CASP6, IKBKB, TRADD, IKBKG, PAK1, NFKBIA, CASP9, MADD, JUN, CRADD, BID, CHUK, NFKBIB, CASP8, BIRC3, MAP4K2, MAP3K14, PAK4, PAK2, PAK6, CASP3, TNFRSF1A, MAP3K1, APAF1, MAPK8, IKBKE, NFKB2, XIAP, FADD, FOS, TRAF2, RIPK1, PAK3, CASP2, PAK7, CYCS, TNF, CASP7, BIRC2 |

**Table S8. Genes involved in the top Ingenuity^®^ canonical pathways**

**LEGENDS TO SUPPLEMENTARY FIGURES**

**Figure S1. Correlations between growth inhibitions by thiopurine drugs among 53 lymphoblastoid cell lines**

**A**. Correlation between growth inhibition by 6-MP 2 µM and by azathioprine 5 µM (r_spearman_=0.95; *P*<0.0001)

**B**. Correlation between growth inhibition by 6-MP 2 µM and by 6-TG 0.5 µM (r_spearman_=0.81; *P*<0.0001). Data of growth inhibition by 6-TG 0.5 µM are available only for 43 cell lines.

**Figure S2. Principal component analysis for the 11 lymphoblastoid cell lines used for the transcriptomic analysis**

#xxxx, cell line ID; R, resistant cell line; S, sensitive cell line.

**Figure S3. Functional downstream *CDKN1A* network**
