# Supplementary figures and images for: Molecular insight into thiopurine resistance: transcriptomic signature in lymphoblastoid cell lines
Source: Genome Med. 2015 Apr 18;7(1):37. doi: 10.1186/s13073-015-0150-6 (PMC4443628; doi:10.1186/s13073-015-0150-6)

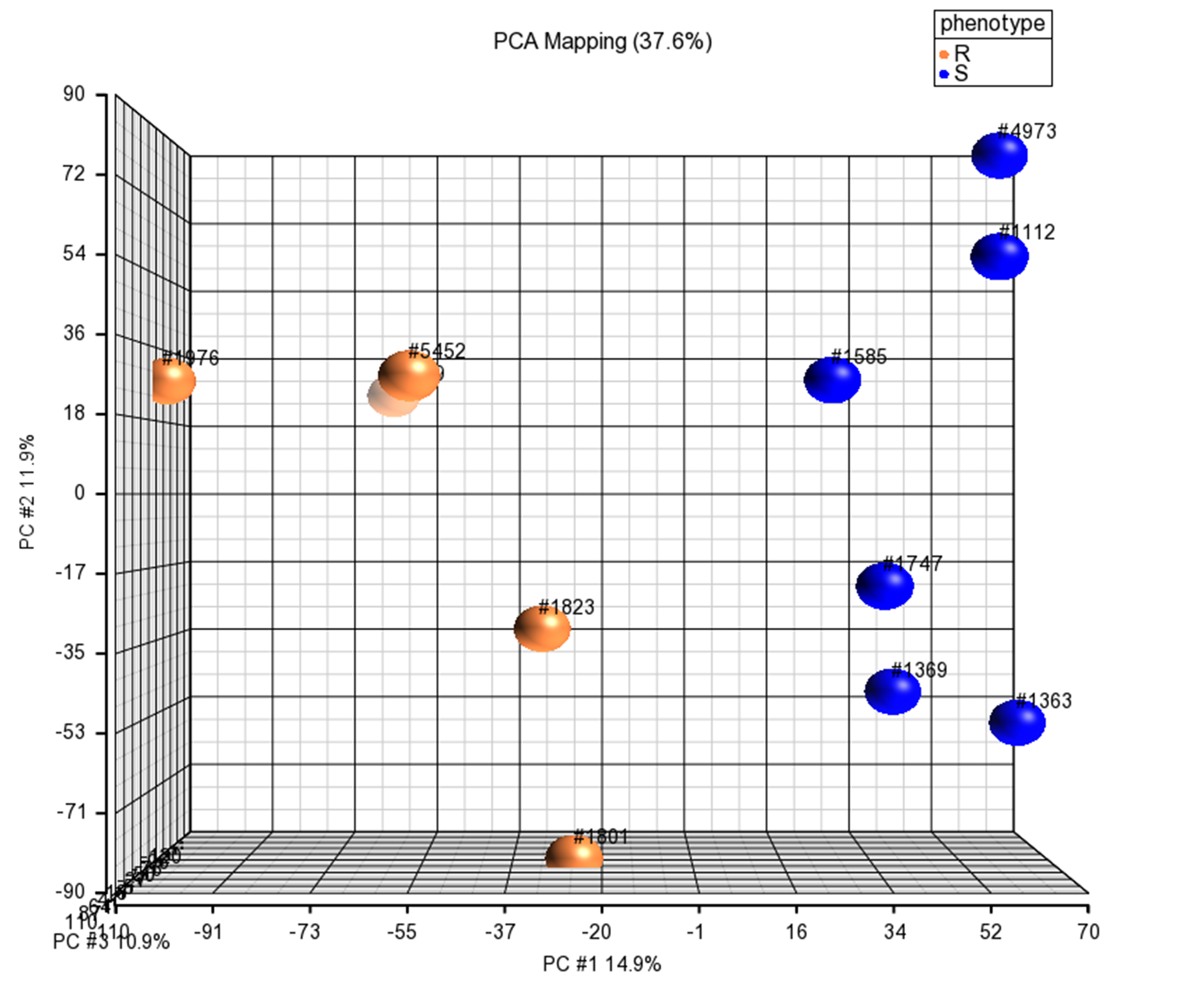

Supplement: Additional file 3: Figure S2. — Principal component analysis for the 11 lymphoblastoid cell lines used for the transcriptomic analysis. #xxxx, cell line ID; R, resistant cell line; S, sensitive cell line. [file 13073_2015_150_MOESM3_ESM.tiff]

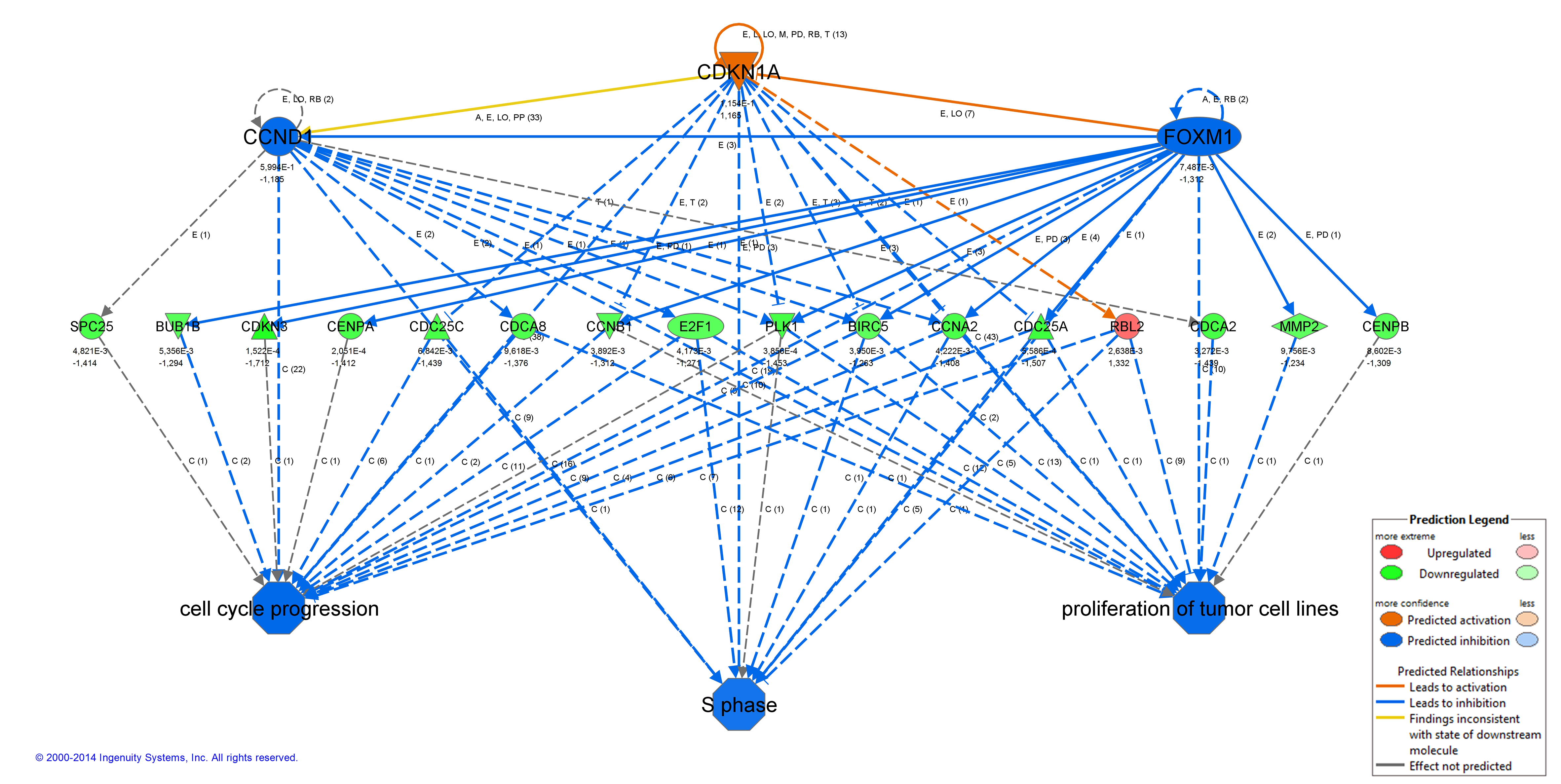

Supplement: Additional file 4: Figure S3. — Functional downstream CDKN1A network. [file 13073_2015_150_MOESM4_ESM.tiff]
